# Inorganic Sodium Solid-State Electrolytes: Progress, Existing Issues, and Solutions Towards High-Performance All Solid-State Batteries

**DOI:** 10.1007/s41918-026-00279-y

**Published:** 2026-02-17

**Authors:** Lingjun Huang, Chun Huang

**Affiliations:** 1 Department of Materials, Imperial College London, London, SW7 2AZ UK; 2 The Faraday Institution, Quad One, Becquerel Ave, Harwell Campus, Didcot, OX11 0RA UK; 3 Research Complex at Harwell, Rutherford Appleton Laboratory, Didcot, OX11 0FA UK

**Keywords:** In-situ/operando techniques, Machine learning, Na solid-state electrolytes, All solid-state batteries, Ionic conductivity, Na dendrite, Mixed-ion strategy, Interface engineering

## Abstract

Na-ion batteries (NIBs) have gained attention as a cost-effective option for large-scale energy storage, offering electrochemical properties similar to lithium-ion batteries (LIBs). To improve safety and energy density, solid-state electrolytes (SSEs) are being incorporated into NIBs, paving the way for high-performance all-solid-state sodium-ion batteries (ASSNIBs). This review summarises recent progress in Na-based SSEs, categorised into oxides, sulfides, and halides, with particular emphasis on their crystal structures, ion conduction mechanisms, and electrochemical performance. We then critically examine the key challenges facing ASSNIBs, including low ionic conductivity, unstable electrode/electrolyte interfaces, and the reliance on rare or costly materials. To gain deeper insights into these issues, we highlight advanced characterisation and modelling techniques, including cryogenic electron microscopy, in-situ/operando characterisation, and machine learning approaches—all of which contribute to understanding Na-ion transport mechanisms and interfacial dynamics more comprehensively, and comparing with conventional electrochemical tests, structural characterisation and modelling methods. Building on these insights, we explore promising strategies such as microstructural design, mixed-ion approaches, and interface engineering to overcome the current limitations in Na SSEs. Finally, we offer perspectives on future research directions to support the rational design and optimisation of Na SSEs, ultimately advancing the development of next-generation ASSNIBs. The advanced characterisation and machine learning methodologies emphasised herein will also prove valuable for broader applications in electrochemical energy storage systems.

**Graphical abstract:**

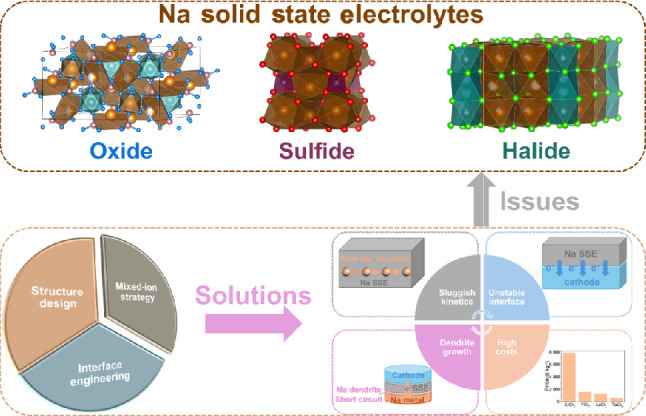

## Introduction

The rapid growth of industrial production and global population has intensified the demand for energy, while existing fossil fuel reserves are insufficient to sustain future needs [1, 2]. Additionally, fossil fuel combustion releases substantial CO_2_, a key driver of climate change [3, 4]. To address this energy shortfall and work toward net-zero CO_2_ emissions, renewable energy sources, such as solar, wind, and nuclear power, have been proposed as viable solutions [5–7]. However, the inherent intermittency of renewables limits their reliability, underscoring the need for efficient, high-capacity energy storage systems [8–10].

Lithium–ion batteries (LIBs) have become the dominant energy storage technology due to their high energy density, long lifespan, wide electrochemical stability window (ESW), and lack of memory effect, making them ideal for mobile electronics and electric vehicles [11–19]. Nonetheless, reliance on lithium, which is often mined in remote or politically sensitive regions, raises concerns about the cost-effectiveness of LIBs for large-scale storage [20, 21]. Furthermore, replacing Li with Na introduces new challenges, including slower ion kinetics, reduced energy density, and greater electrode volume expansion, attributed to Na’s larger ionic radius, higher atomic weight, and lower redox potential compared to Li [22–24].

Na–ion batteries (NIBs) offer a promising alternative, with sodium carbonate available at low cost ($400 per ton for sodium carbonate) and a favourable redox potential ($${{E}}_{\mathrm{(}{\mathrm{Na}}^{+}\mathrm{/Na)}}^{\Theta}\text{=-2.71 }{\mathrm{V}}$$ vs. standard hydrogen electrode (SHE)) [25, 26]. NIBs operate on a similar “rocking-chair” mechanism as LIBs, retaining many advantages while potentially reducing costs [27, 28].

However, they encounter issues similar to those of LIBs, such as safety risks due to Na dendrite penetration through organic liquid electrolytes (OEs), which can cause short circuits, especially when Na metal is used as the anode to boost energy density [29–32]. Furthermore, replacing Li with Na introduces new challenges, including slower ion kinetics, reduced energy density, and greater electrode volume expansion, attributed to the larger radius (1.06 Å (1 Å = 1 × 10^−10^ m)), higher weight (23 g mol^−1^) and potential (–2.71 V vs. SHE) of Na compared to those of Li (with radius of 0.76 Å, weight of 6.9 g mol^−1^ and potential of –3.05 V vs. SHE) [25, 27, 33, 34].

To address these issues, extensive research has explored solid-state electrolytes (SSEs) to enable the development of all-solid-state batteries (ASSBs) [29, 35–39]. With non-flammable properties and wide ESW, ASSBs offer a pathway to safely incorporate high-capacity Na metal anodes (with theoretical specific capacity of 1 166 mAh g^−1^ and reduction potential of –2.71 V vs. SHE) without compromising safety [40–42].

The history of Na-ion conductors dates back to the 1960s, beginning with the discovery of high-temperature Na^+^ conductors like *β*”-alumina-type NaAl_11_O_17_ [43]. Later, the NASICON-type superionic conductors and other sulfide and halide Na^+^ conductors expanded the family of Na superionic conductors [44–46]. By utilising these materials as SSEs, high-performance all-solid-state Na–ion batteries (ASSNIBs) have rapidly become a focus of research.

This review summarises recent progress in Na SSEs, focusing on the development of oxide, sulfide, and halide systems, with particular emphasis on their crystal structures, ion conduction mechanisms, and electrochemical properties. Key challenges, such as low ionic conductivity, unstable electrode/SSE interfaces, and high material costs, are critically examined. We highlight the role of advanced characterisation and modelling techniques, including advanced techniques such as cryogenic transmission electron microscopy (cryo-TEM), in-situ/operando techniques, and machine learning-accelerated modelling, in advancing the understanding of Na-ion transport mechanisms and interfacial dynamics in ASSNIBs, and comparing with conventional electrochemical tests, structural characterisation and modelling methods. Finally, based on the insights obtained from the advanced characterisation and modelling techniques, we discuss promising improvement strategies to enhance the performance of Na SSEs and corresponding ASSNIBs. Overall, this review aims to clarify the key design principles for high-performance Na SSEs and offer guidance for the development of next-generation ASSNIBs.

## The State-of-the-Art Na SSEs

**Fig. 1:**
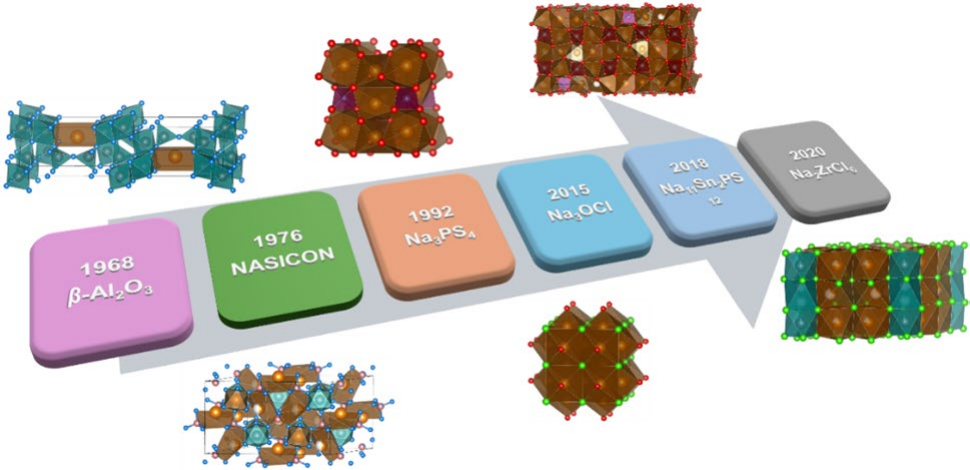
Development and categories of Na SSEs

Based on the types of anions, Na SSEs are categorized into oxide, sulfide, and halide-based electrolytes. This section presents recent developments in these Na SSEs, with a primary focus on their crystal structures, ionic conductivities, and Na-ion conduction mechanisms. Figure 1 shows the development of different types of Na SSE materials.

### Oxide-based Na SSEs

Oxide-based Na SSEs have a long history spanning nearly 50–60 years, marking the beginning of research into Na-ion conductors [43, 44, 47]. They can be categorised by crystal structure into *β*-alumina and NASICON types, which are discussed in detail.

#### β-Alumina SSEs

*β*-Alumina (*β*-Al_2_O_3_) was initially investigated in the study of ternary Ca_2_O–Al_2_O_3_–MgO system [48], and it was originally recognized as an allotropic form of *α*-Al_2_O_3_ until the discovery of trace alkali oxide in its component. Following this, Na-*β*-Al_2_O_3_ was identified as a novel alkali aluminate (Na_2_O·11Al_2_O_3_) [49, 50], leading to further development of Na-*β*”-Al_2_O_3_ (Na_2_O·5Al_2_O_3_) in research of binary Na_2_O–Al_2_O_3_ system [51]. The study of fast Na^+^-conduction in *β*-Al_2_O_3_ began in 1967, marking a milestone in Na SSE development [43].

**Fig. 2:**
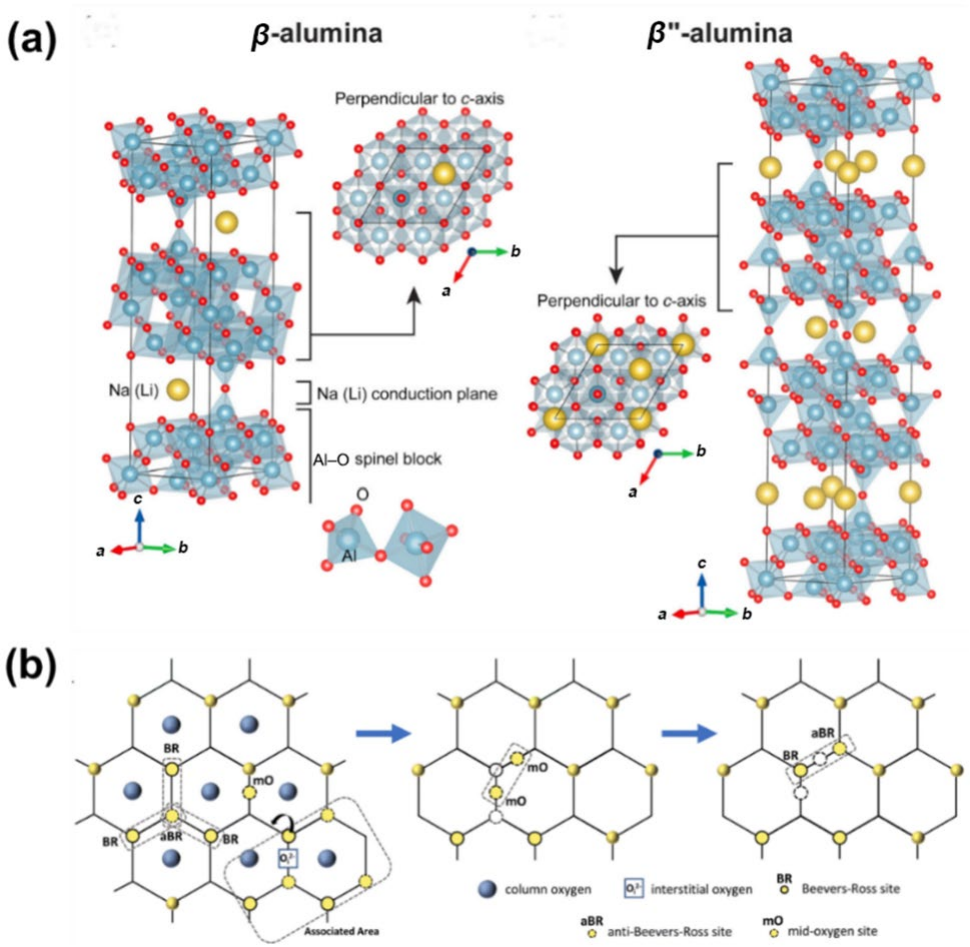
**a** Crystal structures of *β*-alumina (left) and *β*”-alumina (right). Reproduced with permission from Ref. [52]. Copyright © 2017, Elsevier. **b** Interstitial ion-conduction mechanism of *β*-alumina. Reproduced with permission from Ref. [27]. Copyright © 2023, Royal Society of Chemistry

Due to compositional differences, Na-*β*-Al_2_O_3_ (Na_2_O·*x*Al_2_O_3_) is divided into two groups, Na-*β*-Al_2_O_3_ (*x* = 8–11) and Na-*β*”-Al_2_O_3_ (*x* = 5–7), as illustrated in Fig. 2a [52–55]. Structurally, *β*-Al_2_O_3_ has a hexagonal lattice with *P6*_*3*_*/mmc* symmetry, whereas *β*”-Al_2_O_3_ exhibits a rhombohedral lattice with ${\mathrm{R}}\bar{3}{\mathrm{m}}$ symmetry. Both *β*-alumina share a layered configuration of conduction and non-conduction planes stacked alternately. The non-conduction planes, termed spinel blocks, consist of tightly packed [AlO_4_] and [AlO_6_] units, while conduction planes are loosely packed O^2−^ with mobile Na^+^ between bonded spinel blocks. In *β*-Al_2_O_3_, the conduction layer acts as a mirror plane between two spinel blocks, whereas *β*”-Al_2_O_3_ features three spinel blocks separated by two conduction layers.

These structural differences create distinct Na^+^ conduction mechanisms in the two *β*-alumina. In *β*-Al_2_O_3_, the conduction planes include three unique Na^+^ sites: Beevers-Ross (BR), mid-oxygen (mO), and anti-Beevers-Ross (aBR). Na^+^ migration is limited at low temperatures, as Na^+^ ions are restricted by interstitial O^2−^, resulting in a complex interstitial conduction mechanism (left panel in Fig. 2b). As temperature rises, Na^+^ gradually escapes this restriction, progressing through three stages (corresponding to left, middle, and right panels in Fig. 2b): (I) occupied BR sites and aBR sites occupied with excessive Na^+^ interstitial form interstitial pairs. (II) interstitial jump is performed by interstitial pairs to mO–mO configuration. (III) random jumps to initial BR–aBR or adjacent BR–aBR configurations [56, 57]. This mechanism leverages interstitial pair synergy to achieve a low ion-diffusion energy barrier [58]. In *β*”-Al_2_O_3_, a higher concentration of BR sites and additional Na-ion vacancies lead to a vacancy-dominated conduction mechanism [59]. With an exceptionally low ion-diffusion energy barrier (0.02 eV) around vacancies, single-crystal *β*ʺ-Al_2_O_3_ demonstrates ultrahigh room-temperature ionic conductivity, reaching 100 mS cm^−1^ [60, 61].

#### NASICON SSEs

Na superionic conductor, commonly known as NASICON, is another promising oxide-based solid electrolyte material due to its superior thermal and chemical stability, broad ESW, and high RT ionic conductivity (0.1 to 1 mS cm^−1^) [62–66]. With a chemical formula of Na_1+*x*_Zr_2_Si_*x*_P_3−*x*_O_12_ (0 ⩽ *x* ⩽ 3), NASICON was first studied by Goodenough and Hong et al. [44, 67]. The three-dimensional NASICON framework consists of interconnected [ZrO_6_] octahedra and [(Si, P)O_4_] tetrahedra. These units connect via corner-sharing to form infinite ribbons along the *c*-axis, with Na^+^ ions located within the framework’s cavities, linked by broad channels that facilitate Na^+^ migration.

**Fig. 3:**
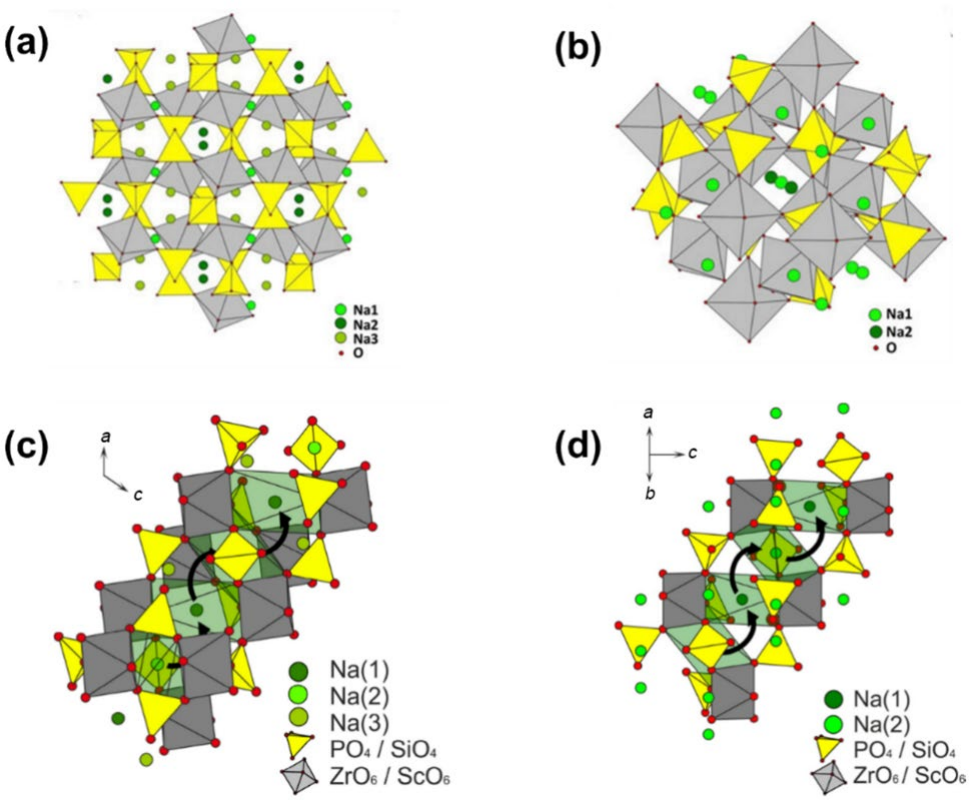
**a** Crystal structures of monoclinic and **b** rhombohedral Na_3_Zr_2_Si_2_PO_12_ [62]. Ion-conduction pathways of **c** monoclinic and **d** rhombohedral Na_3+*x*_Sc_*x*_Zr_2−*x*_(SiO_4_)_2_(PO_4_). The black arrows represent the Na^+^ transport routes. Reproduced with permission from Ref. [73]. Copyright © 2016, American Chemical Society

NASICONs adopt different crystal structures depending on the *x* value. When *x* ranges from 0 to 1.8, NASICON crystallizes in a rhombohedral structure with *R*-3*c* symmetry; as *x* increases to between 1.8 and 2.3, it transitions to a monoclinic structure (*C*2/*c* symmetry), then returns to rhombohedral as *x* continues to increase [68–72]. The monoclinic and rhombohedral NASICONs are depicted in Fig. 3a and b, respectively. Using Na_3+*x*_Sc_*x*_Zr_2−*x*_(SiO_4_)_2_(PO_4_) as an example, Na^+^ migration pathways in both structures are shown in Fig. 3c and. In the monoclinic case, Na^+^ occupies three distinct sites located at the Wyckoff positions 4d, 4e, and 8f, while the rhombohedral NASICON contains two Na^+^ sites at 6b and 18e. Despite the different Na^+^ site locations, the ion-migration pathways are similar, as the rhombohedral NASICON is a slightly twisted variant of the monoclinic structure.

The bulk ionic conductivity of NASICON is determined by three main factors. First, the concentration of charge carriers, which is directly proportional to ionic conductivity. Second, the number of vacant Na^+^ sites, which enable Na^+^ to hop between sites. The ideal ratio of occupied Na sites to vacancies in NASICON has been shown to be 3.4:0.6 [65]. Third, the size of the bottleneck in the Na^+^ transport pathway plays a critical role, as Na^+^ ions must pass through two oxygen triangles from one site to another, making the bottleneck size crucial in determining NASICON’s ionic conductivity.

### Sulphide-Based Na SSEs

Since the development of the glass–ceramic Na-ion conductor Na_3_PS_4_, sulfide-based Na SSEs have gained significant attention for their excellent ion-conducting properties. The subsequent discovery of Na_11_SnP_2_S_12_, a Na^+^ conductor with a structure similar to Li_10_GeP_2_S_12_, introduced new concepts for Na superionic conductors [74, 75]. This section discusses two main categories of sulfide-based Na SSEs: Na_3_PS_4_-type and Na_11_SnP_2_S_12_-type SSEs.

#### Na_3_PS_4_-Type SSEs

**Fig. 4:**
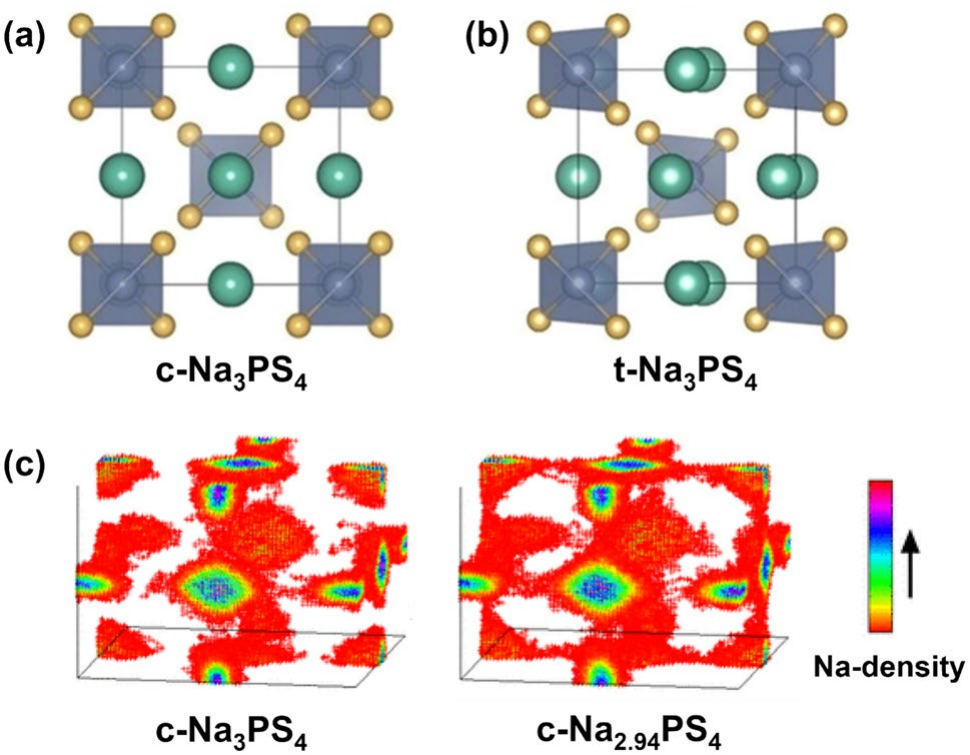
**a** Crystal structures of c-Na_3_PS_4_ and **b** t-Na_3_PS_4_. Reproduced with permission from Ref. [76]. Copyright © 2019, American Chemical Society. **c** Na^+^ distribution map of c-Na_3_PS_4_ (left panel) and c-Na_2.94_PS_4_ (right panel) obtained from 100 ps by MD simulation at 525 K. Reproduced with permission from Ref. [82]. Copyright © 2016, American Chemical Society

Na_3_PS_4_ solid electrolyte exists in two crystalline phases: tetragonal-Na_3_PS_4_ (t-Na_3_PS_4_) and cubic-Na_3_PS_4_ (c-Na_3_PS_4_), as shown in Fig. 4a and b, with c-Na_3_PS_4_ displaying ${\mathrm{I}}\bar{4}{\mathrm{3}}{\mathrm{m}}$ symmetry and t-Na_3_PS_4_ exhibiting ${\mathrm{P}}\bar{4}{{2}}_{1}{\mathrm{c}}$ symmetry [76]. The two distinct phases mainly differ in two ways: (I) PS_4_^3−^ forms a body-centred cubic (bcc) lattice in c-Na_3_PS_4_, while in t-Na_3_PS_4_, they undergo slight distortion along the *c*-axis with rotation along the [111] direction; (II) c-Na_3_PS_4_ has one Na^+^ site while t-Na_3_PS_4_ has two, originating from its tetragonal distortion.

This structural variation impacts Na^+^ conductivity, with t-Na_3_PS_4_ achieving ~ 10^−3^ mS cm^−1^ at 50 °C and c-Na_3_PS_4_ reaching 0.1 mS cm^−1^ at room temperature [77–79]. Theoretical calculations, however, suggest minimal intrinsic conductivity difference between these structures [80]. Pair distribution function (PDF) analysis indicates a presence of microstructural tetragonal features within c-Na_3_PS_4_, suggesting that the enhanced ionic conductivity is likely due to ball-milling-induced defects, which create pressure, strain, and activate Na^+^ migration via vacancy effects [81, 82]. To interrogate the vacancy effect, 2% vacancies are introduced in molecular dynamics (MD) simulations to observe the Na distribution from 100 ps. In Na_3_PS_4_, only Na^+^ vibration can be seen while diffusion hardly happens (left panel in Fig. 4c). On the contrary, Na^+^ mobility grows significantly in Na_2.94_PS_4_ (right panel in Fig. 4c), indicating the vacancy effect in Na^+^ transport.

Beyond P and S, researchers have started to explore alternative elements, such as Sb, thus yielding Na_3_SbS_4_. Similar to Na_3_PS_4_, Na_3_SbS_4_ possesses both cubic and tetragonal phases, and c-Na_3_SbS_4_ has better ion-conducting behaviour than t-Na_3_SbS_4_ (2.8 to 1.77 mS cm^−1^) [83, 84]. Novel synthetic methods, including aqueous-solution synthesis and hard and soft acid–base (HSAB) approaches, have been developed for Na_3_SbS_4_ to improve particle size control and scalability [85–88]. Besides, Se substitution for S in Na_3_PSe_4_ has also achieved a high ionic conductivity of 1.16 mS cm^−1^ [89]. Density functional theory (DFT) studies predict that Na_3_PX_4_ material (X = O, S, Se) with alternative stoichiometries, such as Na_7_P_3_S_11_ and Na_7_P_3_Se_11_, may surpass 10 mS cm^−1^ in RT ionic conductivity, though synthesis challenges remain due to thermal instability [90].

#### Na_11_Sn_2_PS_12_-Type SSEs

Since the development of the Li^+^ superionic conductor Li_10_GeP_2_S_12_, which achieves high RT ionic conductivity (12 mS cm^−1^) [91], researchers have sought to explore Na-analogues with similar properties. Initial theoretical studies on Na_10_GeP_2_S_12_ predicted a high RT ionic conductivity of 4.7 mS cm^−1^ in its distorted tetragonal structure [92]. Experimentally, Na_10_SnP_2_S_12_ demonstrated a lower RT ionic conductivity of 0.4 mS cm^−1^, hindered by impurities such as Na_2_S, P_2_S_5_ and Na_3_PS_4_ [93].

**Fig. 5:**
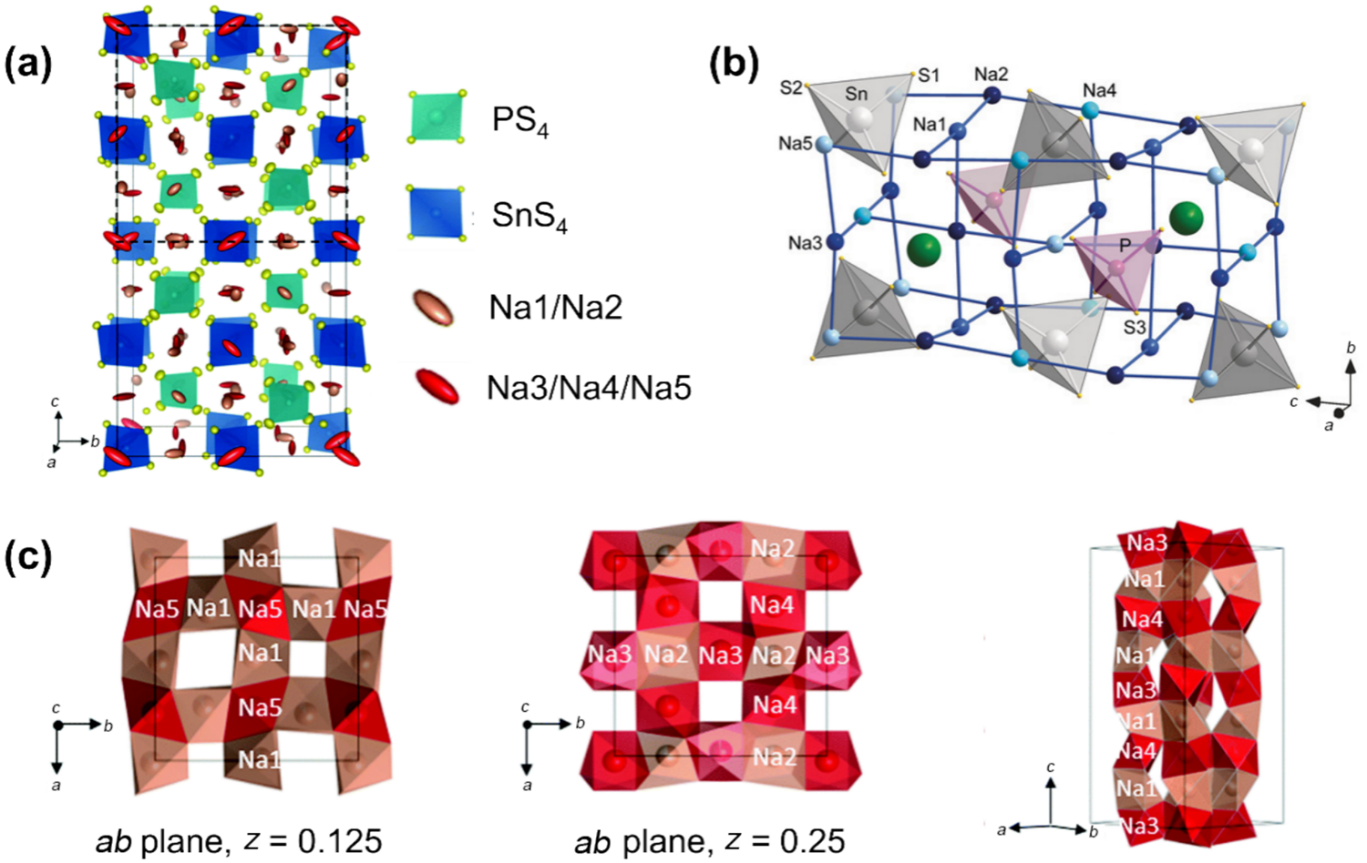
**a** Lattice framework of Na_11_Sn_2_PS_12_. Reproduced with permission from Ref. [94]. Copyright © 2018, Royal Society of Chemistry. **b** 3D Na^+^-conduction network of Na_11_Sn_2_PS_12_. Reproduced with permission from Ref. [95]. Copyright © 2018, Wiley–VCH. **c** Illustrations of Na^+^ transport chains Na(1)–Na(5) (left panel), Na(3)–Na(2) and Na(4)–Na(2) (middle panel), along with Na(4)–Na(1)–Na(3)–Na(1) (right panel). Rose ellipsoids represent Na(1) and Na(2), while red ellipsoids represent Na(3), Na(4) and Na(5), respectively. Reproduced with permission from Ref. [94]. Copyright © 2018, Royal Society of Chemistry

In 2018, the discovery of Na_11_Sn_2_PS_12_ marked significant progress, establishing a Na superionic conductor analogous to Li_10_GeP_2_S_12_ with RT ionic conductivity above 1 mS cm^−1^ [94, 95]. X-ray diffraction (XRD) patterns reveal a tetragonal structure with *I*41/*acd* symmetry in Na_11_Sn_2_PS_12_ (Fig. 5a), forming a 3D chessboard framework with [SnS_4_] and [PS_4_] units that provide Na^+^ diffusion pathways along the *c*-axis. Figure 5b shows the Na^+^ sites within the lattice, each coordinated by four neighbouring Na^+^ ions, resembling the NaCl-type NbO structure with a 25% vacancy ratio in a 2 × 1 × 1 NaCl supercell. Correspondingly, 12 Na^+^ occupy sites alongside two [SnS_4_]^4−^ and one [PS_4_]^3−^ unit in Na_11_Sn_2_PS_12_, leaving one remaining vacancy, indicated by a green sphere in Fig. 5b.

Na^+^ diffusion in Na_11_Sn_2_PS_12_ proceeds along interconnected chains in both [010] and [001] directions, as shown in Fig. 5c. Three Na^+^ sites (Na(3), Na(4), and Na(5)) are nearly fully occupied (with occupancies of 0.96, 0.97, and 0.95, respectively), while two sites (Na(1) and Na(2)) are partially occupied (with occupancies of 0.87 and 0.78), facilitating rapid Na^+^ transport. In Fig. 5c, Na^+^ migration pathways cross at *z* = 0.125 along Na(1)–Na(5) chains and at *z* = 0.25 along Na(3)–Na(2) and Na(4)–Na(2) chains, forming diffusion channels along the *c*-axis. Ab initio molecular dynamics (AIMD) simulations also confirm that these five Na sites and the distributed Na^+^ vacancies enhance Na^+^ mobility [96]. Based on the ion-transport theory, non-stoichiometric composition, Na_10.8_Sn_1.9_PS_11.8_, was developed, achieving a RT ionic conductivity of 0.67 mS cm^−1^ [97].

Liquid-phase synthesis techniques have been successfully applied to Na_11_Sn_2_PS_12_, similar to those used for Na_3_SbS_4_. By mixing raw materials in 1,2-dimethoxyethane and subjecting them to subsequent sintering, researchers obtained liquid-phase-synthesized Na_11_Sn_2_PS_12_ with particle sizes around 200 nm [98]. This synthesized material exhibits a RT ionic conductivity of 0.173 mS cm^−1^, comparable to that of its ball-milled counterpart, which has a conductivity of 0.216 mS cm^−1^. In another study, Na_11_Sn_2_PS_12_ was synthesized using a solvent mixture of 1,2-ethylenediamine (EDA)–1,2-ethanedithiol (EDT), resulting in a RT ionic conductivity of 0.10 mS cm^−1^ [99]. Additionally, the liquid-phase synthesis approach was extended to the nanosized Na_10_GeP_2_S_12_-based electrolyte, Na_10_SnSb_2_S_12_, which demonstrates a RT ionic conductivity of 0.52 mS cm^−1^ [100].

To expand the family of Na_11_Sn_2_PS_12_-type solid electrolyte materials, researchers began investigating the effects of substituting P with other group 15 elements, such as Sb, or adjacent group elements like Si. Both Na_4−*x*_Sn_1−*x*_Sb_*x*_S_4_ (0.02 ⩽ *x* ⩽ 0.33) and Na_4_Sn_0.67_Si_0.33_S_4_ displayed crystal structures similar to Na_11_Sn_2_PS_12_ (*I*41/*acd* space group), and exhibit relatively high RT ionic conductivities of 0.51 mS cm^−1^ for Na_3.75_Sn_0.75_Sb_0.25_S_4_ and 1.6 mS cm^−1^ for P-doped Na_3.75_[Sn_0.67_Si_0.33_]_0.75_P_0.25_S_4_ [101–103]. Furthermore, a selenide-based quaternary material, Na_11.1_Sn_2.1_P_0.9_Se_12_, was identified, achieving a high RT ionic conductivity of 3.0 mS cm^−1^ [104]. The influence of the anion lattice on Na^+^ transport in Na_11_Sn_2_PnX_12_ (Pn = P, Sb; X = S, Se) has also been studied [105]. In highly conductive Na_11_Sn_2_PS_12_ and Na_11_Sn_2_PSe_12_, the rotation of [PX_4_]^3−^ units is observed, whereas such rotation is restricted in the low-conductive Na_11_Sn_2_SbS_12_. This restriction on [SbS_4_]^3−^ rotation is believed to hinder Na^+^ transport due to a lack of fluctuation that widens the ion-migration bottlenecks, as seen with [PX_4_]^3−^.

The ionic conductivities, activation energy, and crystal structures of oxide and sulfide-based Na SSEs are summarised in Table 1.

**Table 1:** Summary of oxide and sulfide-based Na SSEs and their characteristics

Material	RT conductivity/(mS cm^−1^)	Activation energy/eV	Crystal structure	References
0.84Na_2_O·0.67MgO·5.2 Al_2_O_3_ (single crystal)	10–180	0.20	Rhombohedral $\left(R\overline{3 }m\right)$	[61]
Na_3_Zr_2_Si_2_PO_12_	1.1	0.60 (Na/SSE interface)	NASICON	[66]
Na_3_PS_4_	4.17 ✕ 10^−3^ (50 °C)	0.42	Tetragonal $\left(P\overline{4}{2 }_{1}c\right)$	[77]
Na_3_PS_4_	0.2	0.28	Cubic $\left(I\overline{4 }3m\right)$	[78]
Na_3_PS_4_	0.46	0.20	Cubic $\left(I\overline{4 }3m\right)$	[79]
Na_3_SbS_4_	2.8	0.06	Cubic $\left(I\overline{4 }3m\right)$	[83]
Na_3_SbS_4_	1.77	0.25	Tetragonal $\left(P\overline{4}{2 }_{1}c\right)$	[83]
Na_3_SbS_4_	3	0.25	Tetragonal $\left(P\overline{4}{2 }_{1}c\right)$	[84]
Na_3_SbS_4_	1.05	0.22	Tetragonal $\left(P\overline{4}{2 }_{1}c\right)$ at RT	[87]
Na_3_PSe_4_	1.16	0.21	Cubic $\left(I\overline{4 }3m\right)$	[89]
Na_7_P_3_O_11_ (theoretical)	0.003	0.535	Triclinic $\left(P\overline{1 }\right)$	[90]
Na_7_P_3_S_11_ (theoretical)	10.97	0.217	Triclinic $\left(P\overline{1 }\right)$	[90]
Na_7_P_3_Se_11_ (theoretical)	12.56	0.213	Triclinic $\left(P\overline{1 }\right)$	[90]
Na_10_GeP_2_S_12_ (theoretical)	4.7	0.20	Distorted tetragonal	[92]
Na_10_SnP_2_S_12_	0.4	0.356	Tetragonal	[93]
Na_11_SnP_2_S_12_	1.4	0.25	Tetragonal $\left(I41/acd\right)$	[94]
Na_11_SnP_2_S_12_	$3.7\pm 0.3$	0.387	Tetragonal $\left(I41/acd\right)$	[95]
Na_10.8_Sn_1.9_PS_11.8_	0.67	0.307	Tetragonal $\left(I41/acd\right)$	[97]
Na_11_SnSb_2_S_12_	$0.56\pm 0.03$	0.34	Tetragonal $\left(I41/acd\right)$	[101]
Na_4−*x*_Sn_1−*x*_Sb_*x*_S_4_ (0.02 ⩽ *x* ⩽ 0.33)	0.2–0.5	0.39 (*x* = 0.40)	Tetragonal $\left(I41/acd\right)$	[102]
Na_4_Sn_0.67_Si_0.33_S_4_	1.23 ✕ 10^−2^	0.56	Tetragonal $\left(I41/acd\right)$	[103]
Na_11.1_Sn_2.1_P_0.9_Se_12_	3.0	0.30	Tetragonal $\left(I41/acd\right)$	[104]

### Halide-Based Na SSEs

Despite initial investigations into halide-based ionic conductors, such as Li_2_ZnCl_4_ and LiI during the 1970s and 1980s, research in this area waned due to their relatively low RT ionic conductivity (less than 0.01 mS cm^−1^) [106, 107]. However, the discovery of anti-perovskite Li_3_OX (X = Cl or Br) in 2012 marked a turning point, as these materials demonstrated ionic conductivities exceeding 1 mS cm^−1^, thereby reigniting interest in halide-based SSEs [108]. Halide-based Na SSEs are classified into two categories based on their central elements: anti-perovskite SSEs and metal-centred SSEs.

#### Anti-perovskite SSEs

Anti-perovskite SSEs with non-metal central elements, represented as Na_3_BA (B denotes divalent anions and A represents halogen anions), exhibit excellent reduction stability but poor oxidation stability, contrasting sharply with their metal-centred counterparts [109, 110].

**Fig. 6:**
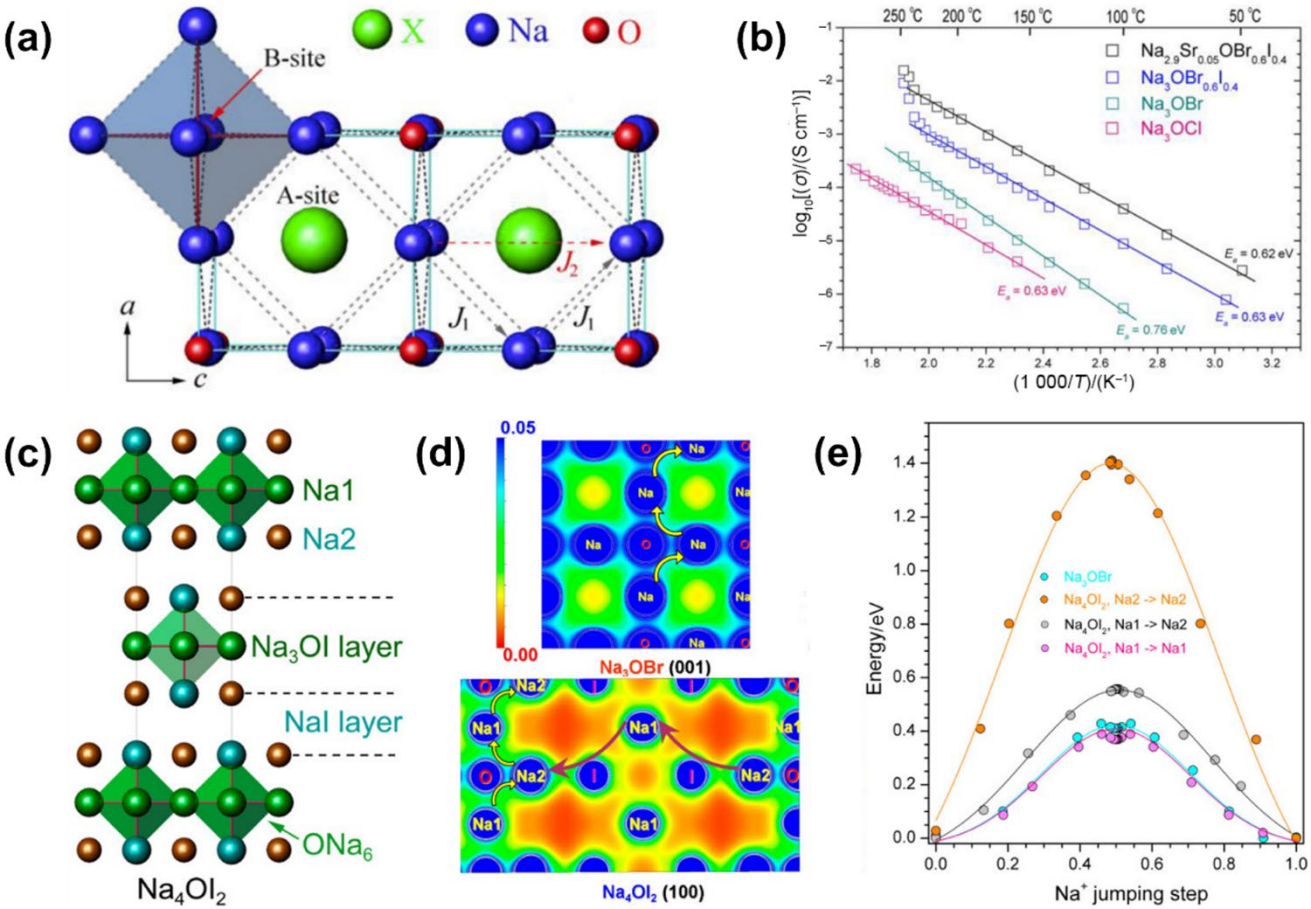
**a** Crystal structure of Na_3_OX (X = Cl, Br, and I). Blue polyhedron and dashed lines represent [ONa_6_] unit and nearest Na-Na distances respectively, while J1 and J2 show the Na-ion migration pathways within lattice. **b** Arrhenius plots for Na_3_OCl, Na_3_OBr, Na_3_OBr_0.6_I_0.4_, and Na_2.9_Sr_0.05_OBr_0.6_I_0.4_. Reproduced with permission from Ref. [111]. Copyright © 2015, Elsevier. **c** Crystal structure of Na_4_OI_2_. **d** Nuclear density distribution maps of (001) plane of Na_3_OBr and (100) plane of Na_4_OI_2_ at 500 K. The arrows demonstrate the favourable ion-migration pathways in Na_3_OBr and Na_4_OI_2_. **e** Na^+^ jumping energies along different diffusion paths. Reproduced with permission from Ref. [112]. Copyright © 2016, American Chemical Society

In 2015, Na-based anti-perovskite SSEs, Na_3_OX (X = Cl, Br, I), were synthesized using a straightforward method involving a mixture of Na, NaOH and NaX [111]. The synthesized materials crystallize in a structure with *Pm* $\bar{3}$ *m* symmetry, where [ONa_6_] units connect through shared corners, while halogen anions occupy 12-coordinated sites (Fig. 6a). For Na^+^ migration, the [101] and [001] directions are identified as key pathways when considering vacancy concentration in this structure. Figure 6b illustrates the Arrhenius plots of four representative Na_3_OX materials, in which ionic conductivities exhibit a trend of ${\sigma}_{{\mathrm{Na}}_{3}{\mathrm{OCl}}}\mathrm{<}{\sigma}_{{\mathrm{Na}}_{3}{\mathrm{OBr}}}\mathrm{<}{\sigma}_{{\mathrm{Na}}_{3}{\mathrm{O}}{\mathrm{Br}}_{0.6}{\mathrm{I}}_{0.4}}\mathrm{<}{\sigma}_{{\mathrm{Na}}_{2.9}{\mathrm{Sr}}_{0.05}{\mathrm{O}}{\mathrm{Br}}_{0.6}{\mathrm{I}}_{0.4}}$. The trend is attributed to two factors: (I) a mismatching effect stemming from the incorporation of larger Br^−^ and I^−^, which creates ample space for Na^+^ hopping through interstitial pathways. (II) an increased vacancy concentration due to a divalent cation substitution, providing effective migration routes for Na^+^. The optimised Na_2.9_Sr_0.05_OBr_0.6_I_0.4_ finally reaches an RT ionic conductivity of 2.78 × 10^−3^ mS cm^−1^.

Another study investigated Na-ion migration pathways in Na_3_OBr and layered Na_4_OI_2_ (with structure shown in Fig. 6c) using temperature-dependent neutron diffraction [112]. In the nuclear density distribution maps (Fig. 6d), it is observed that Na^+^ hops among nearest neighbour positions within [ONa_6_] unit, while Br^−^ is excluded in Na_3_OBr. Conversely, in Na_4_OI_2_, although Na^+^ at Na1 sites follows a similar migration mechanism in the *ab*-plane as in Na_3_OBr, a distinct ion-migration pathway along the *c*-axis is noted. Here, Na^+^ at the Na2 sites exhibit elliptic nuclear density, overlapping with the I^−^ nuclear densities polarized toward Na2 ions, indicating that I^−^ play a crucial role in Na^+^ transport along *c*-axis rather than O^2−^. To further validate the proposed ion-migration mechanism, theoretical simulations were conducted to calculate the Na^+^ jumping energies (Fig. 6e). The results demonstrate that Na^+^ migration energy in Na_3_OBr is isotropic, with a value of 0.34 eV, while Na_4_OI_2_ shows three different energy values, the largest corresponding to Na2 − Na2 migration, almost threefold greater than Na1 − Na2 and Na1 − Na1 migration. Therefore, Na-ion transport is regarded anisotropic within the [Na_3_OI] layer of Na_4_OI_2_.

DFT simulations have been employed in the study of anti-perovskite SSEs to elucidate Na-ion migration mechanisms. These studies reveal that the structure significantly influences Na^+^ transport kinetics, providing a theoretical framework for the rational design of high ion-conducting anti-perovskite SSEs [113–115]. In addition to using halogens as *A*-site elements, other anions such as BH_4_^−^, NH_2_^−^ and AlF_4_^−^ also present viable alternatives for *A* sites in anti-perovskite structures. These anions have demonstrated RT ionic conductivities exceeding 1 mS cm^−1^, as their larger sizes facilitate the creation of wide channels for Na^+^ transport [111, 116–118].

#### Metal-Centred SSEs

Since the development of Li^+^ conductors Li_3_YCl_6_ and Li_3_YBr_6_ by Asano et al. [119], the potential of halide-based SSEs with metal-centred elements (Na_*a*_M_*b*_X_*c*_, M and X are metal cation and halogen anion, respectively) has been extensively studied. Compared to oxide and sulfide-based SSEs, the weaker Coulombic interactions between monovalent X^−^ (X = F, Cl, Br, I) and Na^+^, along with higher electronegativity and larger ionic radii of halogen, confer halide-based SSEs with fast ion-conducting capabilities, high oxidative stability, and excellent deformability, making them promising electrolyte materials [119–121].

**Fig. 7:**
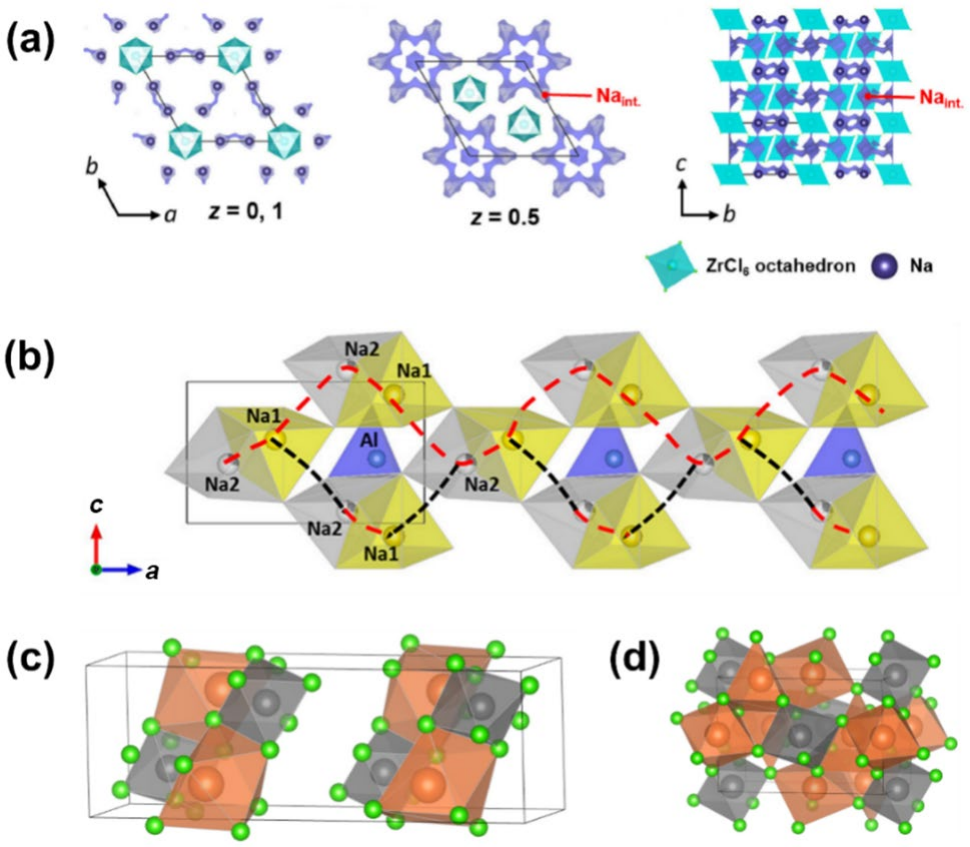
BVEL results of Na-ion migration channels in **a** Na_2_ZrCl_6_ Reproduced with permission from Ref. [122]. Copyright © 2021, Elsevier. and **b** [010] direction of NaAlCl_4_. Reproduced with permission from Ref. [46]. Copyright © 2022, American Chemical Society. Crystal structures of **c** NaTaCl_6_ and **d** Na_3_YBr_6_. Orange, grey and green balls represent Na^+^, transition metal ions (Ta in **c** and Y in **d**), and halide ions (Cl in **c** and Br in **d**)

A cost-effective Na-ion conductor, Na_2_ZrCl_6_ was developed via mechanochemical preparation, demonstrating an ultrahigh oxidative potential up to 5 V (vs Na/Na^+^) [122]. Also, the ball-milled Na_2_ZrCl_6_ (BM-Na_2_ZrCl_6_) exhibits a high RT ionic conductivity of 0.018 mS cm^−1^, three orders of magnitude higher than the heat-treated Na_2_ZrCl_6_ after ball-milling (HT-Na_2_ZrCl_6_). This phenomenon parallels previous studies of Li_3_YCl_6_ and Li_2_ZrCl_6_, where increased crystallinity due to heat treatment reduced ionic conductivity [119, 123]. XRD analysis revealed that BM-Na_2_ZrCl_6_ exhibits *P* $\bar{3}$ *m*1 symmetry, with broad Bragg reflections indicating low crystallinity. Bond valence energy landscape (BVEL) calculations visualized Na-ion diffusion pathways (Fig. 7a). In the *ab*-plane at *z* = 0, Na^+^ migrates between adjacent Na1–Na1 sites, while six Na^+^ interstitial sites form ribbon-shaped paths with low activation energy (0.26 eV) at *z* = 0.5, but they are only short-term diffusion pathways because it is difficult for Na^+^ to migrate to the adjacent ribbons. Although the distance between Na1 is too large for direct hopping, the interstitial sites facilitate migration along the *c*-axis. Therefore, Na1–Na int.–Na1–Na int. along the [001] direction emerges as favourable pathway for Na_2_ZrCl_6_.

Another cost-effective and oxidatively stable SSE, orthorhombic NaAlCl_4_ (*P*2_1_2_1_2_1_ symmetry), was identified [46]. Similar to Na_2_ZrCl_6_, ball-milled NaAlCl_4_ (BM-NaAlCl_4_) exhibits a relatively high RT ionic conductivity of 3.9 × 10^−3^ mS cm^−1^, attributed to its low crystallinity from ball-milling. Combining X-ray Rietveld refinement with BVEL calculations, three Na-ion diffusion channels are revealed, including crossing rectangular faces (I) between face-sharing prisms and (II) between corner-sharing prisms (labelled with red dashed lines in Fig. 7b), along with migrating through (III) triangular and rectangular faces between corner-sharing prisms (black dashed lines in Fig. 7b). These modes collectively form two-dimensional Na-ion migration pathways in the *ac*-plane (Fig. 7b).

Despite satisfactory properties such as high oxidation stability and low cost, Na_2_ZrCl_6_ and NaAlCl_4_ still present challenges for use in ASSNIBs owing to their low ionic conductivity. Therefore, efforts have shifted towards enhancing the ionic conductivity of metal-centred Na halide SSEs, exemplified by the successful exploration of NaTaCl_6_. Mechanochemical synthesis of NaTaCl_6_, adopting a monoclinic *P*2_1_/*n* symmetry in its crystalline form, has achieved an ultrahigh RT ionic conductivity of 4 mS cm^−1^ (Fig. 7c) [124]. This remarkable performance is rooted in a structural transition from a low-conductive crystalline phase to a highly disordered amorphous network induced by extended high-energy ball milling. The amorphous phase comprises a dynamically restructured poly-(TaCl_6_) framework that enables rapid Na^+^ transport through an open and weakly coordinated halide matrix. A suite of advanced characterisation unravelled the underlying conduction mechanism. XRD patterns reveal a gradual disappearance of long-range order with increasing milling time, consistent with the transition to a predominantly amorphous state. Concurrently, Arrhenius plots show a decrease in activation energy from nearly 0.41 eV to 0.30 eV, indicating reduced energy barriers for Na^+^ migration. Na K-edge X-ray absorption spectroscopy (XAS) suggests a disruption in the local symmetry around Na ions and weaker Na–Cl interactions in the amorphous state, evident from the attenuated pre-edge features and sharper spectral edges. Meanwhile, Ta L3-edge extended X-ray absorption fine structure (EXAFS) and wavelet transform (WT) analysis reveal increasing distortion and proximity of TaCl_6_ octahedra, supporting the formation of a compact, percolating network conducive to ion mobility. Raman spectra further corroborate the breakdown of ordered TaCl_6_ dimers, with diminished vibrational features signalling enhanced structural disorder. Solid-state ^23^Na MAS nuclear magnetic resonance (NMR) measurements detect a shift to more negative chemical shifts and signal broadening in the amorphous sample, indicative of a more heterogeneous and electron-rich Na environment. PDF analysis shows increased medium-range Cl–Cl correlations, revealing that [TaCl_6_] units become more tightly clustered as disorder develops, which contributes to creating interconnected migration channels for Na^+^ ions. In summary, these findings illustrate that the exceptional ionic conductivity of amorphous NaTaCl_6_ arises not from conventional vacancy diffusion but from a combination of disrupted electrostatic interactions, high polyhedral connectivity, and a softened, glassy matrix [124]. Similarly, NaNbCl_6_ with *P*2_1_/*c* symmetry, achieved an ionic conductivity of 3.1 × 10^−3^ mS cm^−1^ at 30 °C [125].

Beyond chloride-based SSEs, bromide-based Na_3_YBr_6_ was also explored [126]. Synthesized via solid-phase reaction between NaBr and YBr_3_, this monoclinic structure (*P*2_1_/*c*, shown in Fig. 7d) exhibits contrasting properties compared to chloride counterparts, achieving a low activation energy of 0.15 eV while maintaining a low ionic conductivity of 4.57 × 10^−5^ mS cm^−1^. Theoretical simulations attribute this phenomenon to the large dimension and vibration of Br^−^, which creates ample diffusion channels for Na^+^, resulting in low activation energy despite poor Na^+^ mobility.

**Fig. 8:**
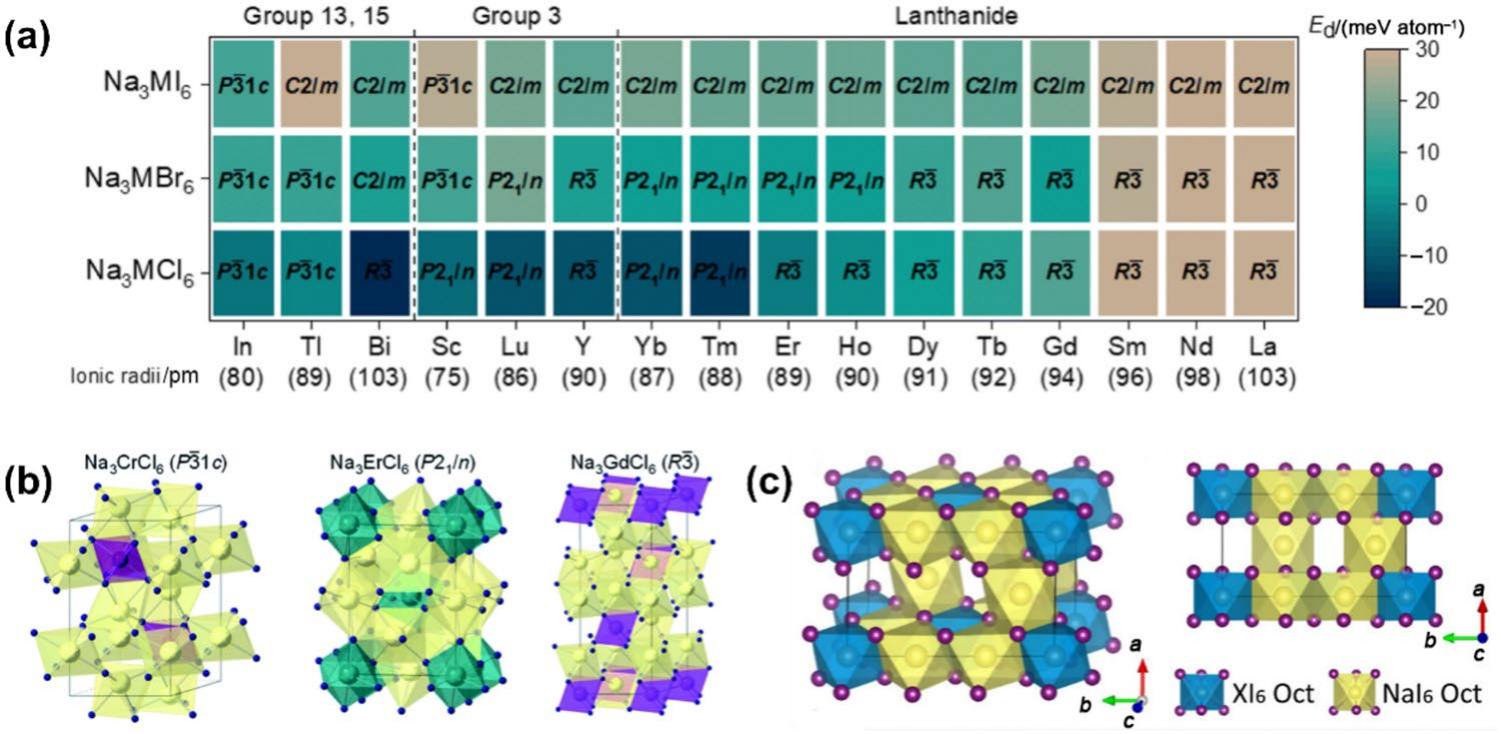
**a** Heat map based on *E*_d_ of Na_3_MX_6_ (X = Cl, Br and I), indicating the relatively stable phase structures of Na_3_MX_6_. Reproduced with permission from Ref. [127]. Copyright © 2022, Royal Society of Chemistry. **b** Crystal structures of Na_3_MCl_6_ (M = Cr, Er, and Gd), in which yellow polyhedra represent [NaCl_6_] unit. Reproduced with permission from Ref. [131]. Copyright © 2021, Royal Society of Chemistry. **c** Crystal structure of *C*2/*m*-Na_3_XI_6_ (X = Sc, Y, La, and In). Yellow, blue and purple balls represent Na, X, and I, respectively. Reproduced with permission from Ref. [132]. Copyright © 2022, American Chemical Society

In addition, theoretical studies were conducted to support the rational design and mechanism investigations of metal-centred halide SSEs. The phase stability of Na_3_MX_6_ was evaluated using decomposition energy (*E*_d_), identifying the most stable crystal structures. Na_3_MX_6_ compounds with *E*_d_ values below 0 are regarded as stable phases [127]. Those with positive *E*_d_ are classified into two groups: phases with *E*_d_ below 25 meV per atom are metastable phases due to entropic effects and kinetic barriers to decomposition, while others are unstable phases that will decompose into NaX and MX_3_. The phase structure and stability of Na_3_MX_6_ are strongly influenced by the types and sizes of M and X. Specifically, (I) when X = Cl or Br, phases with *P* $\bar{3}$ 1*c*, *P*2_1_/*n* and *R* $\bar{3}$ symmetries are favoured, while *C*2/*m* phases demonstrate greater stability and higher ionic conductivity (about 0.1 mS cm^−1^) when X = I. (II) Na_3_MCl_6_ phases are generally more stable than Na_3_MBr_6_ and Na_3_MI_6_, reflecting the enhanced stability associated with smaller X anions. (III) Na_3_MX_6_ phases featuring large M cations (*r*_M_ > 95 pm) such as Sm, Nd and La, tend to be unstable. These principles are shown in Fig. 8a. Besides, a series of studies also focused on specific chloride-based Na halides, including *C*2/*m*- and *P* $\bar{3}$ *m*1-Na_3_MCl_6_ (M = In and Sc) [128], *P*321-Na_3_YCl_6_ and *C*2-Na_3_YBr_6_ [129], Na_*x*_M_*y*_Cl_6_ (M = La–Sm) with UCl_3_-type structure [130], along with *P*3 $\bar{1}$ *c*-Na_3_CrCl_6_, *P*2_1_/*n*-Na_3_ErCl_6_ and *R* $\bar{3}$-Na_3_GdCl_6_ (structures shown in Fig. 8b) [131]. The predicted materials exhibit high RT ionic conductivities, even exceeding 1 mS cm^−1^, providing fundamental guidelines for developing novel Na halide SSEs.

Utilising first-principles and AIMD simulations, iodide-based Na_3_XI_6_ (X = Sc, Y, La, and In) were systematically investigated. Featuring 3D octahedral − tetrahedral − octahedral Na-ion migration pathways, Na_3_XI_6_ exhibit both structural stability (*C*2/*m* space group, shown in Fig. 8c) and high RT ionic conductivities of 0.36, 0.35, and 0.20 mS cm^−1^ for X as Sc, Y, and La, respectively [132]. Furthermore, ion-conducting mechanisms in Na_3_YI_6_ with different crystal structures were explored [133]. Similar to *C*2/*m*-Na_3_YI_6_, octahedral-tetrahedral-octahedral channels also dominate the ion-migration paths in *P* $\bar{3}$ *m*1 and *P* $\bar{3}$ 1*c*-Na_3_YI_6_, resulting in RT ionic conductivities of 0.18, and 9.1 × 10^−3^ mS cm^−1^, respectively. The deficient Na-ion transport kinetics in *P* $\bar{3}$ 1*c*-Na_3_YI_6_ are attributed to the blockage of 1D Na-ion migration pathways by adjacent [YI_6_] octahedra.

The ionic conductivities, activation energy, and crystal structures of halide-based Na SSEs are summarised in Table 2.

**Table 2:** Summary of halide-based Na SSEs and their characteristics

Material	RT conductivity/ (mS cm^−1^)	Activation energy/eV	Crystal structure	Reference
Na_3_OCl	About 2.00 ✕ 10^−2^ (200 ℃)	0.63	Cubic $\left(Pm\overline{3 }m\right)$	[111]
Na_3_OBr	About 7.94 ✕ 10^−2^ (200 ℃)	0.76	Cubic $\left(Pm\overline{3 }m\right)$	[111]
Na_3_OBr_0.6_I_0.4_	9.80 ✕ 10^−2^ (160 ℃)	0.63	Cubic $\left(Pm\overline{3 }m\right)$	[111]
Na_2.9_Sr_0.05_OBr_0.6_I_0.4_	2.78 ✕ 10^−3^	0.62	Cubic $\left(Pm\overline{3 }m\right)$	[111]
Na_3_OBr	1.62 ✕ 10^−2^ (150 ℃)	0.680	Cubic $\left(Pm\overline{3 }m\right)$	[112]
Na_4_OI_2_	7.74 ✕ 10^−2^ (200 ℃)	0.646	Cubic $\left(Pm\overline{3 }m\right)$	[112]
Na_2_ZrCl_6_	0.018	0.40	Trigonal $\left(P\overline{3 }m1\right)$	[122]
NaAlCl_4_	3.9 ✕ 10^−3^	0.42	Orthorhombic $\left(P{2}_{1}{2}_{1}{2}_{1}\right)$	[46]
NaTaCl_6_	4	0.30	Monoclinic $\left(P{2}_{1}/n\right)$	[124]
NaNbCl_6_	3.1 ✕ 10^−3^	0.48	Monoclinic $\left(P{2}_{1}/c\right)$	[125]
Na_3_YBr_6_	4.57 ✕ 10^−5^	0.15	Monoclinic $\left(P{2}_{1}/c\right)$	[126]
Na_3_InCl_6_ (theoretical)	2.83	0.27	Monoclinic $\left(C2/m\right)$	[128]
Na_3_InCl_6_ (theoretical)	11.54	0.20	Trigonal $\left(P\overline{3 }m1\right)$	[128]
Na_3_ScCl_6_ (theoretical)	0.66	0.33	Monoclinic $\left(C2/m\right)$	[128]
Na_3_ScCl_6_ (theoretical)	3.09	0.26	Trigonal $\left(P\overline{3 }m1\right)$	[128]
Na_3_YCl_6_ (theoretical)	0.77	0.30	Trigonal $\left(P321\right)$	[129]
Na_3_YBr_6_ (theoretical)	0.44	0.32	Monoclinic $\left(C2\right)$	[129]
NaLa_0.95_Ta_0.43_Cl_6_	1.4	–	Hexagonal $\left(P{6}_{3}/m\right)$	[130]
Na_3_CrCl_6_ (theoretical)	2 ✕ 10^−4^	0.57	Trigonal $\left(P\overline{3 }1c\right)$	[131]
Na_3_ErCl_6_ (theoretical)	2 ✕ 10^−5^	0.65	Monoclinic $\left(P{2}_{1}/n\right)$	[131]
Na_3_GdCl_6_ (theoretical)	2 ✕ 10^−5^	–	Trigonal $\left(R\overline{3 }\right)$	[131]
Na_3_ScI_6_ (theoretical)	0.36	0.33	Monoclinic $\left(C2/m\right)$	[132]
Na_3_YI_6_ (theoretical)	0.35	0.32	Monoclinic $\left(C2/m\right)$	[132]
Na_3_LaI_6_ (theoretical)	0.20	0.37	Monoclinic $\left(C2/m\right)$	[132]
Na_3_YI_6_ (theoretical)	0.18	0.351	Trigonal $\left(P\overline{3 }m1\right)$	[133]
Na_3_YI_6_ (theoretical)	9.1 ✕ 10^−3^	0.454	Trigonal $\left(P\overline{3 }1c\right)$	[133]

## Existing Challenges of Na SSEs

**Fig. 9:**
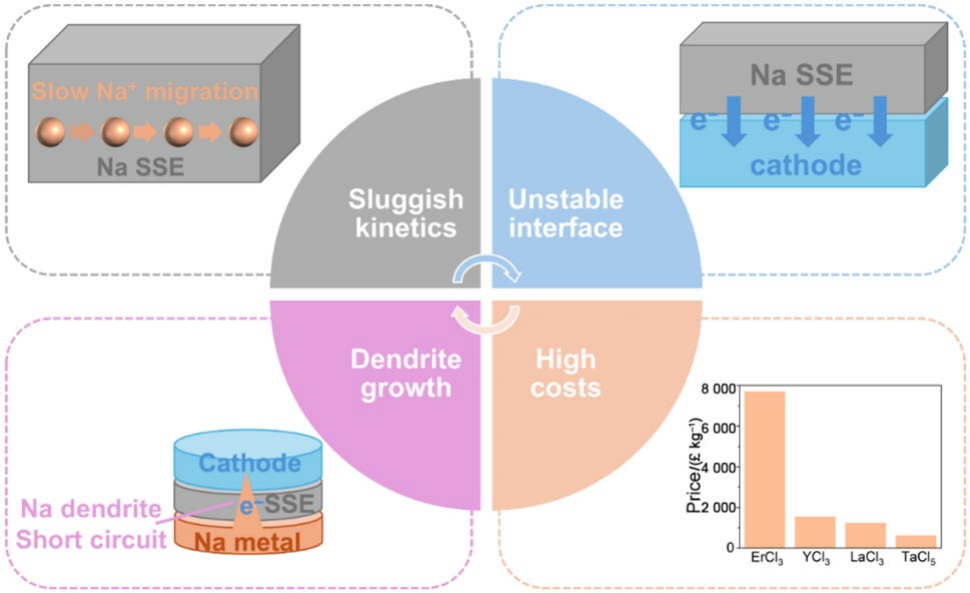
Existing issues of Na SSEs. Cost data originated from Ref. [136]

Ideal SSEs share several common properties: (I) high ionic conductivity (> 1 mS cm^−1^) and low electronic conductivity (< 10^−10^ S cm^−1^), (II) a wide ESW (> 5 V vs. Na^+^/Na), (III) chemical stability and compatibility with anode and cathode materials, (IV) excellent mechanical properties to resist dendrite propagation, (V) high thermal stability, allowing operation over a wide temperature range, (VI) low toxicity and non-flammability, and (VII) cost-effectiveness in sourcing raw materials and synthesis [27, 29, 30, 134, 135]. Despite rapid development in all three types of Na SSEs, many challenges remain. Figure 9 shows the existing challenges of Na SSEs such as slow Na^+^ migration in the SSE materials, the unstable interface between the electrode and SSE, Na dendrite growth, and relatively high costs of the SSE materials. This discussion focuses on three common issues, including low ionic conductivity, unstable electrode/electrolyte interface, and high costs.

### Low Ionic Conductivity

For the practical application of SSEs, their RT ionic conductivities must exceed 1 mS cm^−1^. However, many Na SSEs, including some NASICON, Na_11_Sn_2_PS_12_-type, anti-perovskite and metal-centred halide SSEs, have yet to meet this requirement [137]. Low ionic conductivity results in sluggish Na^+^ transport kinetics, which hinders fast charge and discharge rates in ASSNIBs. Additionally, high internal resistance contributes to increased ohmic polarisation, ultimately reducing Coulombic efficiency (CE) [138, 139].

### Unstable Electrode/SSE Interface

In interfacial stability analysis, both the Na anode/SSE and cathode/SSE interfaces should be taken into consideration.

For the cathode/SSE interface, mismatches in ESW and space charge effects contribute to interfacial instability [140]. When the working potential of the cathode overtakes the upper limit of ESW, parasitic reactions will occur at the interface, leading to the formation of insulating by-products that hinder electronic transfer [141–143]. Additionally, the space charge effect, resulting from differences in chemical potentials between ion-conductors, introduces significant resistance. Due to the chemical potential gap, Na^+^ will transport from SSE to cathode, and electrons will combine with Na^+^ until a new thermodynamic equilibrium is established. Continuous Na^+^ diffusion creates a Na-deficient region in the SSE, which further impedes Na^+^ transport [144–146].

**Fig. 10:**
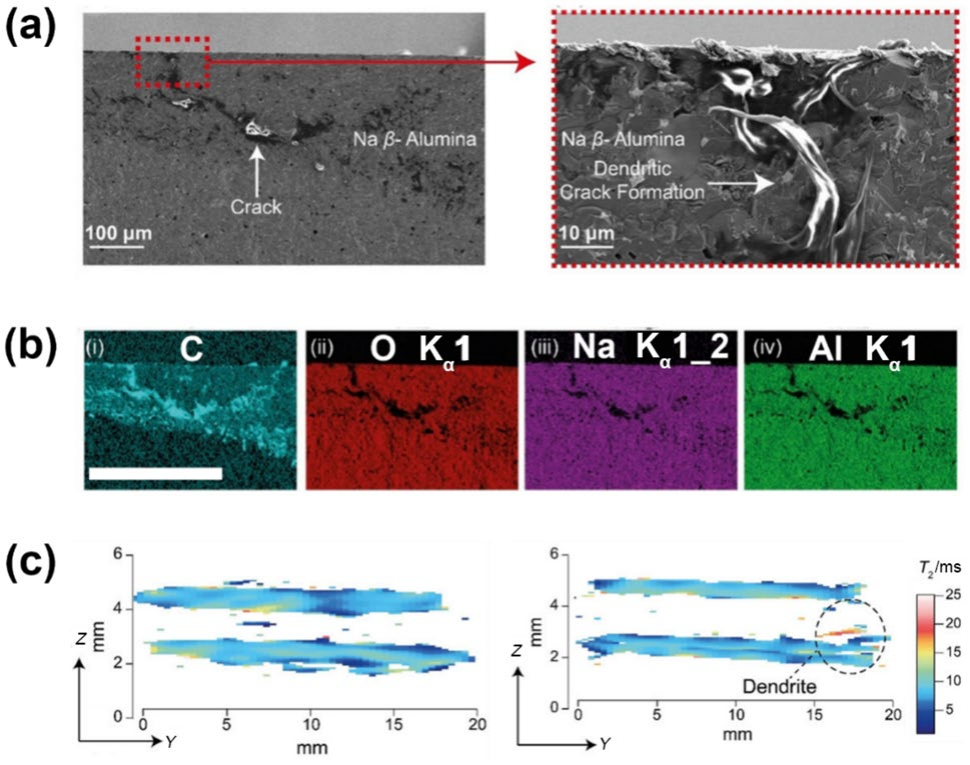
**a** SEM images indicating Na dendritic crack. **b** Corresponding EDS images demonstrating elemental distributions of C, O, Na, and Al. **c** *T*_2_ weighted contrast maps of pristine cell (left panel) and short-circuiting cell (right panel) [155]

For the Na/SSE interface, few SSEs exhibit thermodynamic stability when in contact with Na metal, with Na dendrite growth being a major concern [147–149]. Similar to dendrite initiation and propagation in Li-based ASSBs [150, 151], ASSNIBs may also experience Na dendrite growth, potentially leading to short-circuiting due to the inhomogeneous flux of Na [30, 152–155]. Using Na-*β*”-Al_2_O_3_ as a model SSE, the behaviour of Na dendrite growth was investigated [155]. Scanning electron microscopy (SEM) images revealed a spallation crack (Fig. 10a), which is thought to fill with Na, supported by weak Na signals in the dendritic area observed through energy-dispersive X-ray spectroscopy (EDS) (Fig. 10b). But an uncertainty is still left as a strong carbon signal was also detected, which is probably attributed to the reaction between external C species and reactive Na. Additionally, *T*_2_-contrasted ^23^Na magnetic resonance imaging (MRI) was employed to monitor Na dendrite growth (Fig. 10c). After 11.5 h, a high-contrast region in the *T*_2_ map of a short-circuiting cell indicated dendritic growth due to the longer *T*_2_ of growing Na nuclei, serving as direct evidence of Na dendrite formation. Another study on Na-*β*”-Al_2_O_3_ SSE highlighted that Na dendrite growth results from a cyclic interaction between crack formation and Na deposition. Stress accumulated during Na deposition promotes crack propagation, which in turn aids in the release of local stress and further encourages Na deposition until short circuits occur [156].

### High Costs

**Fig. 11:**
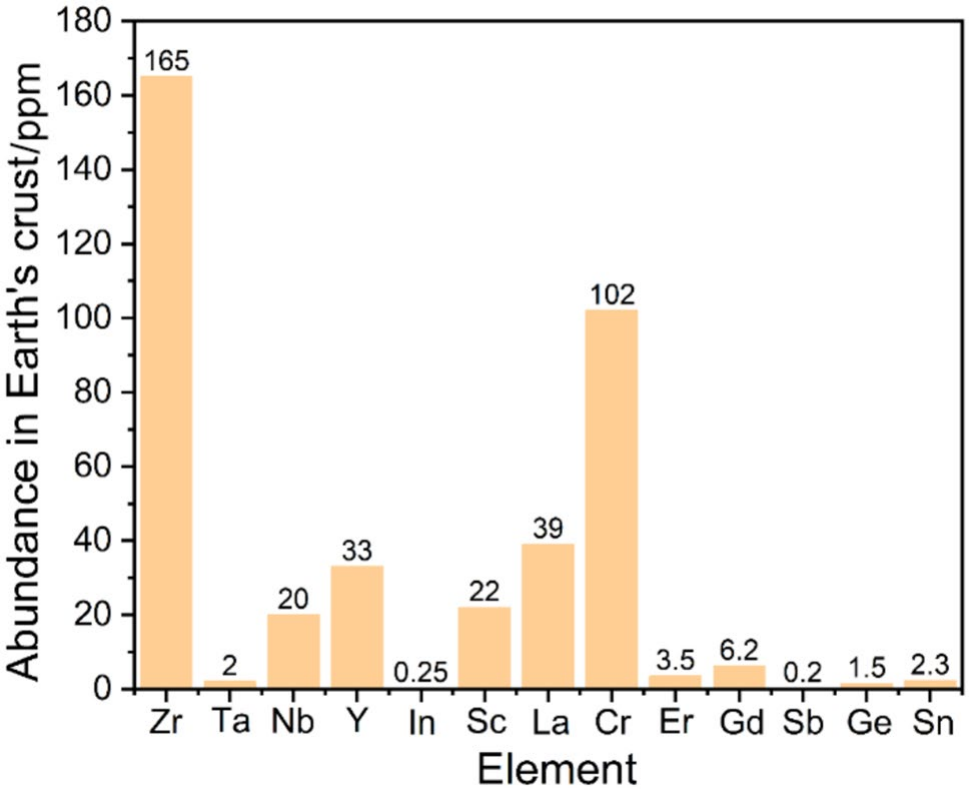
Abundance of common metal elements used in Na SSEs in Earth’s crust. Data originated from Ref. [158]

The cost-effectiveness of Na SSEs primarily depends on two key factors: the cost of raw materials and the associated synthesis processes [109, 123]. For ASSBs to become commercially viable, the total costs for both materials and processing should ideally be maintained below $10 m^−2^ [157]. By substituting Li with Na, a notable reduction in raw material cost has already been achieved [25]. However, introducing less abundant elements (such as Y, In, Sc and Lu) can dramatically elevate raw material expenses, occasionally surpassing $1 000 kg^−1^ [123]. Figure 11 summarises the abundance of selected metal elements commonly employed in Na SSEs. It is evident from the figure that many of these metals exhibit relatively low crustal abundance, often less than 100 μmol mol^−1^ (1 μmol mol^−1^ = 1 ppm), with exceptions such as Zr (165 ppm) and Cr (102 ppm). For contextual comparison, non-metal elements frequently used in battery electrolytes, such as phosphorus (1 050 ppm) and sulfur (350 ppm), possess considerably higher abundance. This inherent scarcity of metal elements directly influences their market prices, consequently increasing the total raw material cost of Na SSEs. For production methodologies, solid-state routes, such as mechanochemical synthesis and high-temperature sintering, are currently the dominant techniques for SSE synthesis due to their straightforward protocols [140]. However, these methods typically require energy-intensive synthesis routes and prolonged processing times, contributing to substantial energy consumption and limiting cost-effectiveness for large-scale manufacturing. Conversely, liquid-phase synthesis approaches offer greater potential for scalability and uniformity at lower processing temperatures, but they are less developed for many Na SSE materials [109].

## Characterisation and Modelling Techniques in Na SSE Studies

To address the aforementioned challenges, it is essential to develop a fundamental understanding of Na^+^ transport behaviour in Na SSE materials, as well as their evolution during cell operation. Achieving this requires the use of a wide range of advanced characterisation and computational modelling tools. In this section, we discuss these approaches as applied in Na SSE research.

### Conventional Characterisation and Modelling Techniques

To investigate ionic transport properties, establish structural frameworks, and predict chemical and electrochemical stability, a range of conventional techniques, spanning electrochemical and structural characterisation, as well as theoretical modelling, have been widely employed.

Electrochemical measurements including cyclic voltammetry (CV), electrochemical impedance spectroscopy (EIS), and direct current (DC) polarisation remain the most widely used tools for assessing electrochemical stability and conductivities of SSE materials [122]. CV is performed by sweeping the potential of a working electrode and recording the resulting current signal, allowing the estimation of the ESW and assessing redox compatibility between SSE materials and both Na metal anode and cathode materials. EIS operates by applying a small alternating voltage and measuring the impedance over a range of frequencies. The results, usually interpreted by equivalent circuit models, allow for quantification of bulk and grain boundary contributions to ionic conductivity. DC polarisation involves applying a constant voltage and observing the steady-state current, where the electronic conductivities can be derived, offering insight into the electronic leakage in SSE materials.

Structural characterisation has primarily been conducted through XRD and, in some cases, neutron diffraction [36]. The principle of XRD is by directing monochromatic X-rays onto the material and detecting the diffraction beams that emerge at specific angles. The resulting diffraction patterns reflect the periodic atomic arrangement and can be used to determine the phase structure and lattice dimensions. Neutron diffraction provides complementary insights, especially for locating light elements like Na and O [159]. Based on these techniques, key features such as crystal structures, lattice parameters, and structural disorder, along with conduction planes and bottleneck geometries responsible for fast Na-ion transport can be visualised. For the study of material microstructures, solid-state NMR spectroscopy and X-ray photoelectron spectroscopy (XPS) have been exploited to investigate local coordination environments and amorphous structure [160]. Solid-state NMR probes the magnetic environment of nuclei such as ^23^Na and yields data on ion site occupation and dynamics, particularly valuable in disordered systems. XPS measures the binding energy of electrons ejected from the surface by X-ray photons, giving surface-sensitive information on chemical composition and electronic states. With the help of these characterisation techniques, atomistic transport information can be obtained, contributing to the theoretical explanation of fast Na-ion conduction mechanisms in designed materials.

On the theoretical side, Bond-Valence Site Energy (BVSE) analysis is useful to visualise Na-ion migration pathways and estimate the energy barriers [122]. This method offers fast, structure-based predictions by identifying low-energy ion-diffusion channels within crystal lattices and can be used to screen ion transport properties across a wide range of structural configurations. DFT has become indispensable for understanding defect chemistry, migration barriers, redox stability, and electronic structure [127, 161]. This method enables the calculation of formation energies of intrinsic and extrinsic defects, including Na vacancies and interstitials, and predicts site preferences and the impact on Na-ion mobility. DFT-derived migration barriers provide a fundamental basis for comparing Na-ion transport across different crystal structures. Furthermore, by analysing the electronic density of states (DOS), DFT helps to assess the bandgap and confirms the electronic insulation behaviour of SSE materials. Additionally, DFT enables ESW evaluation through thermodynamic decomposition energy calculations, which help to estimate compatibility with electrode materials. AIMD provides time-resolved insights into Na-ion diffusion at finite temperatures, particularly in disordered or glassy systems [82, 96, 161]. AIMD captures collective atomic motions and local rearrangements that drive diffusion, offering a mechanistic explanation for high ionic conductivity even in the absence of long-range structural order. These simulations track Na-ion trajectories over time, quantify mean square displacement, and derive self-diffusion coefficients that correlate with experimental conductivity values. For example, AIMD studies could demonstrate how compositional tuning, such as partial oxygen substitution or the inclusion of soft lattice formers, can modify the flexibility of the matrix, which in turn affects Na-ion mobility by altering the energy landscape and coordination environments.

**Fig. 12:**
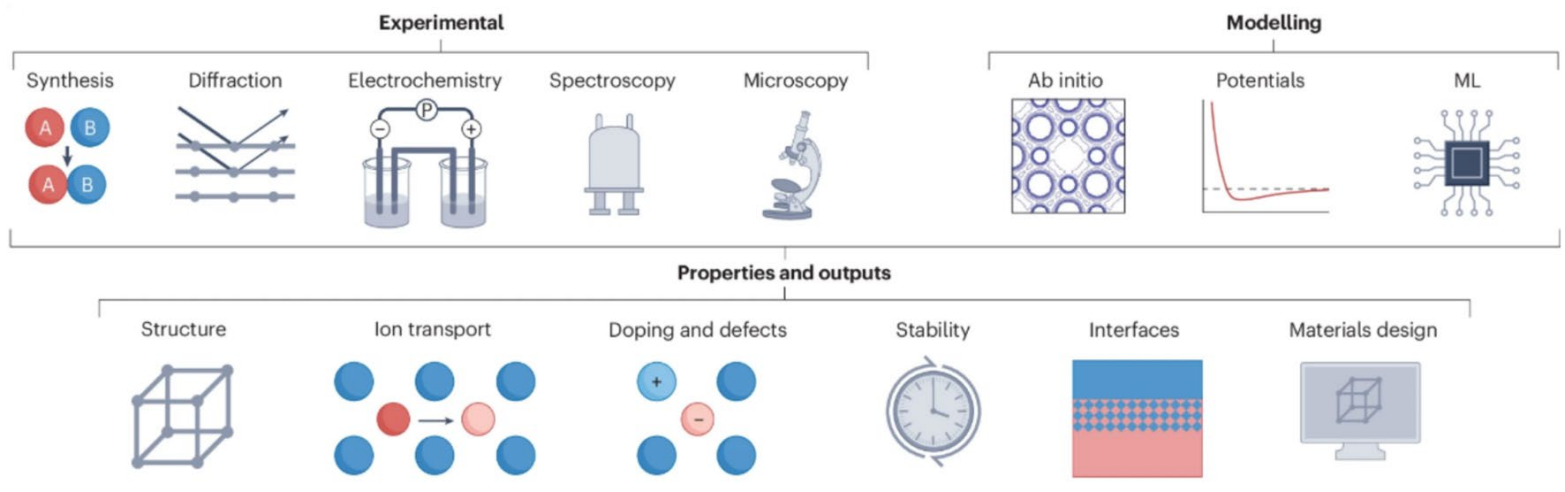
Schematic illustration of characterisation and modelling techniques used in the studies of Na SSE properties. Reproduced with permission from Ref. [161]. Copyright © 2025, Springer Nature

In summary, these techniques have collectively advanced the understanding of structure–property relationships, including both the chemical and electrochemical properties, in Na SSEs (Fig. 12). These characterisation and modelling techniques offer complementary insights, from experimentally determined structures and properties to theoretically predicted behaviours and mechanisms, forming a robust foundation for the design and optimisation of Na SSEs and corresponding next-generation ASSNIBs.

### Advanced Characterisation and Modelling Techniques

To capture spatially resolved Na-ion transport, real-time interfacial evolution, and localised degradation, advanced characterisation approaches are required. This section highlights three such advanced techniques—cryo-electron microscopy, in-situ/operando methods, and machine learning-accelerated modelling—that provide deeper and more comprehensive insights into the behaviour of Na SSEs under realistic operating conditions.

#### Cryo-electron Microscopy

Cryo-electron microscopy is an advanced characterisation method capable of providing crucial evidence of electrode/SSE interfacial evolution down to the atomic scale resolution. In a recent study of cryo-TEM characterisation on the Na–K/NASICON interface in ASSNIBs, it was identified that various crystalline structures, such as Na_2_O, Na_4_P_2_O_7_, and Na_2_ZrSi_3_O_10_, formed by the reaction between anode and NASICON SSE and distributed within an amorphous matrix. The results demonstrated that the heterogeneous SEI structure supports efficient Na-ion transport at the anode/SSE interface and significantly inhibits further side reactions, thus suppressing dendritic growth [162]. Compared to conventional TEM, a key advantage of cryo-TEM is its capability to directly capture the interfacial structures with high precision, which is friendly with amorphous or partially decomposed interfacial layers; the advanced characterisation technique offers insights beyond what conventional characterisation methods can achieve.

**Fig. 13:**
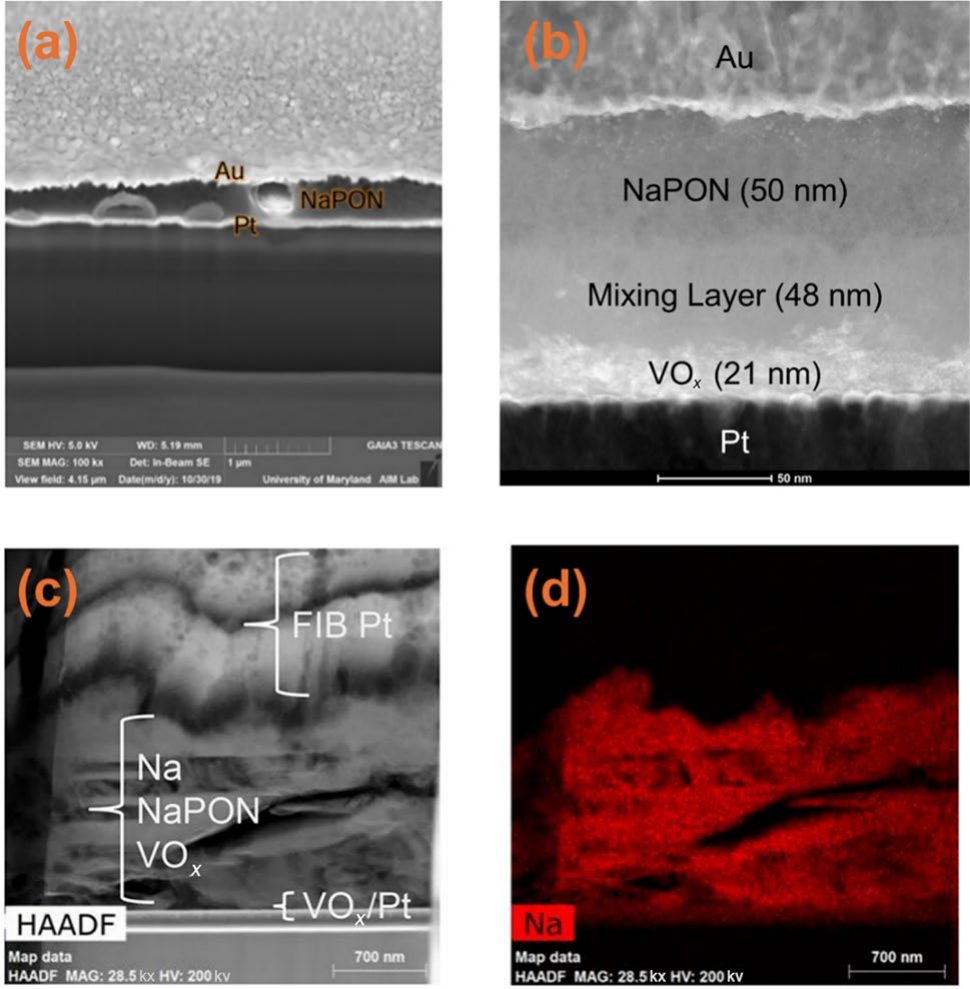
**a** Electron-beam damage of NaPON SSE during focusing when obtaining SEM images by conventional SEM which is mitigated by cryogenic conditions. **b** Cryo-STEM image of NaPON/VO_*x*_/Pt stack, clearly showing three-layer structure. **c** High-angle annular dark-field-STEM (HAADF-STEM) image of Na/NaPON/VO_*x*_/Pt stack and corresponding **d** EDS image of Na. Reproduced with permission from Ref. [163]. Copyright © 2023, American Chemical Society

Similarly, cryogenic scanning transmission electron microscopy (cryo-STEM) also presents unique benefits by effectively preserving beam-sensitive materials under analysis, significantly reducing electron beam-induced artifacts and structural damage common in standard SEM imaging obtained at room temperature (Fig. 13a) [163]. This feature enables reliable nanoscale characterisation of sensitive battery interfaces, making it especially powerful for understanding reactive Na-based systems. Applying cryo-STEM to an ASSNIB composed of a sodium phosphorus oxynitride (NaPON) electrolyte and a vanadium oxide VO_*x*_ cathode, researchers successfully resolved multiple nanoscale layers at their interface. Specifically, cryo-STEM clearly distinguished a pristine NaPON electrolyte layer, an intermixed chemical layer and the underlying VO_*x*_ cathode layer (Fig. 13b). This chemically mixed interlayer originated from cathode/SSE interactions occurring during cell preparation and was further intensified by the thermal deposition of Na metal as revealed in cryo-STEM cross-sectional image (Fig. 13c) and corresponding elemental mapping of Na (Fig. 13d), which show widespread Na distribution throughout these intermixed regions. These interfacial reactions and corresponding by-products significantly impact the electrochemical behaviour of ASSNIBs, in which a capacity decay and increased impedance was observed upon cycling. The detailed structural and compositional insights provided by cryo-STEM analysis clearly associate the declined electrochemical performance with the observed interfacial evolution [163].

In summary, cryo-TEM and cryo-STEM techniques not only enable the visualisation of electrode/SSE interfaces but also provide deep understanding of interfacial evolution and degradation mechanisms. These functions can further guide the interfacial engineering in the construction of ASSNIBs. Nonetheless, cryo-TEM/STEM also have some limitations. For example, the narrow field of view and complex sample preparation process may lead to site-specific bias, and structural interpretation may also be complex due to overlapping signals. Furthermore, integration of cryo-TEM with complementary techniques such as EDS and electron energy loss spectroscopy (EELS) as combined advanced characterisation could potentially expand their capabilities towards correlating local structural properties and chemical reactions.

#### In-Situ and Operando Techniques

While ex-situ techniques offer useful insights into battery materials, their post-mortem nature limits the ability to capture dynamic processes such as phase transitions or intermediate states during cycling [164, 165]. Moreover, exposure to air and moisture can alter sensitive electrodes, making it difficult to accurately assess interfacial reactions or valence changes. Compared to ex-situ characterisation methods, advanced characterisation of in-situ and operando techniques offer distinct advantages by enabling real-time tracking of morphological, chemical, and electrochemical changes during cell operation, enabling a more accurate understanding of structural evolution and its impact on electrochemical performance. These methods are particularly valuable for revealing transient states, intermediate phases, and dynamic interfacial behaviour that cannot be captured post-mortem, making them indispensable for mechanistic studies of dendrite formation, phase transitions, or interfacial layer formation and growth. For operando measurements, the materials or devices are performed under normal operating conditions, which allows continuous, real-time monitoring of chemical and structural evolution under active electrochemical cycling.

**Fig. 14:**
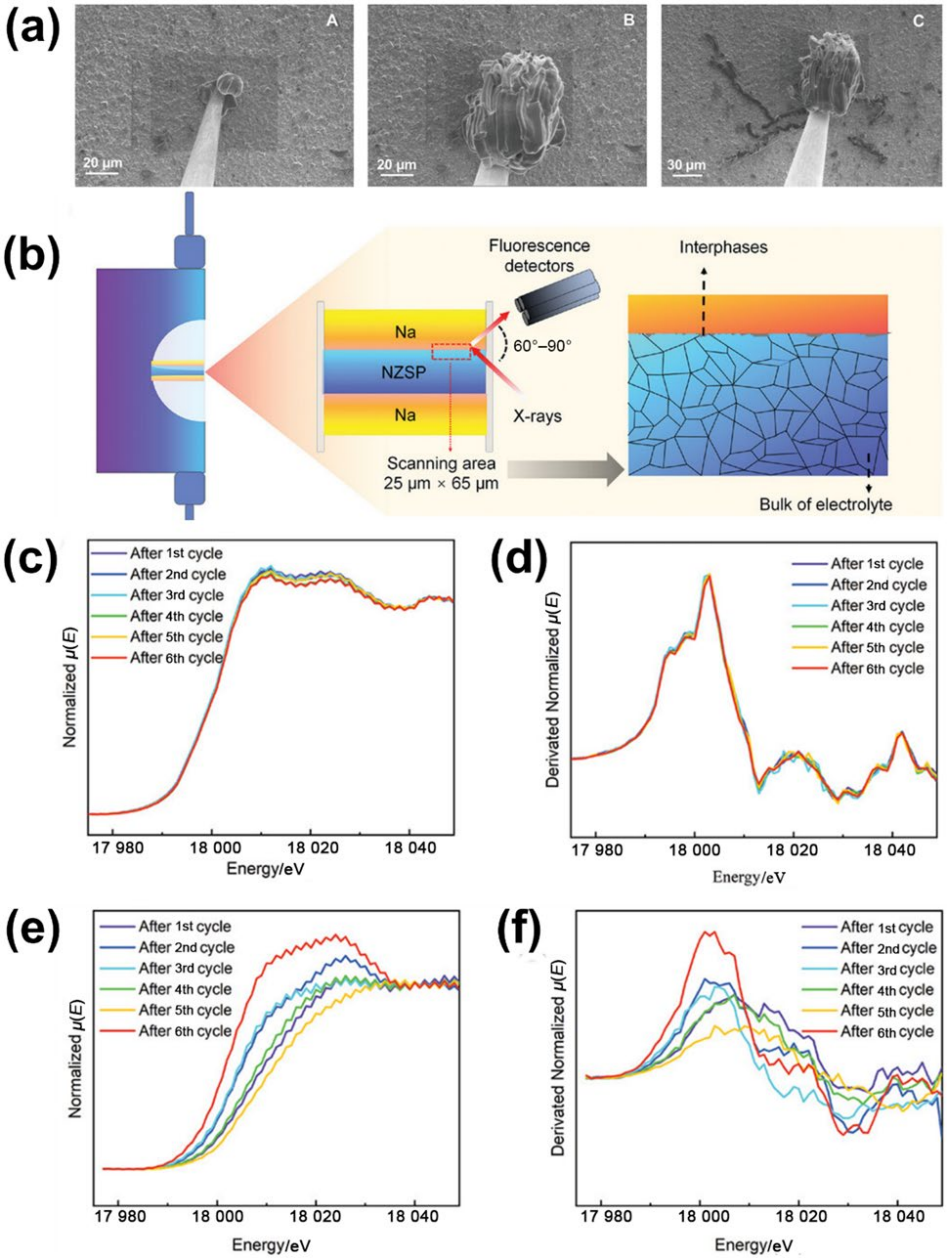
**a** In-situ SEM images of microelectrode evolution with rising applied voltage [166]. **b** Schematic illustration of symmetric Na|Na_3_Zr_2_Si_2_PO_12_|Na cell used for in-situ characterisation. XANES spectra of Zr K-edge, along with corresponding first derivatives of **c, d** Na_3_Zr_2_Si_2_PO_12_ and **e, f** interphases formed at Na/Na_3_Zr_2_Si_2_PO_12_ interface during cycling. Reproduced with permission from Ref. [170]. Copyright © 2023, American Chemical Society

In-situ characterisation, which means “in the natural or original position or place”, has been exploited in probing the complex morphological and electrochemical evolution occurring at the interface between Na SSEs and electrodes. In a representative study, in-situ SEM was combined with a Na microelectrode configuration to investigate dendrite growth behaviour at the Na|Na_3.4_Zr_2_Si_2.4_P_0.6_O_12_ interface [166]. This method enables real-time visualisation of Na deposition under operational current densities, capturing the lateral growth of dendritic filaments along the ceramic surface rather than penetration through the bulk. Apart from the initial two voltage steps at 5 and 10 mV (Fig. 14a, left panel), a slight current increase is observed, likely due to expansion of working electrode area and enhanced Na deposition. The subsequent polarisation steps up to 45 mV yield stable current responses, suggesting predominantly vertical Na growth. Even at 50 mV held for 5 min (Fig. 14a, middle panel), growth remains vertical. However, increasing the bias to 70 mV triggers the rapid emergence of laterally oriented Na filaments, as shown in the right panel of Fig. 14a. Critically, by applying a thin Na-salt layer to the Na_3.4_Zr_2_Si_2.4_P_0.6_O_12_ surface prior to assembly, atmospheric interactions will be modulated and dramatically alters interfacial dynamics. As a result, symmetric Na|Na_3.4_Zr_2_Si_2.4_P_0.6_O_12_|Na cells exhibited a remarkable enhancement in critical current density, achieving values up to 14 mA cm^−2^. The in-situ SEM imaging directly linked dendritic morphology to interfacial chemistry and pressure effects, revealing that growth was initiated at high-current point contacts where voids formed due to mismatched ion flux and self-diffusion rates.

Complementary insights have been provided by other in-situ techniques targeting interfacial stability and degradation. In a study of in-situ Raman spectroelectrochemistry, chemical decomposition of Na_3_SbS_4_ SSE at the anode/SSE interface was monitored, by which the formation of a multicomponent interphase including Na_2_S, Sb_2_S_7_^4−^ and NaSb was observed during Na_3_SbS_4_ reduction at the interface [167]. The accumulation of these products over cycling was correlated with increased cell resistance and irreversible capacity loss. In another work, in-situ XPS was used to investigate the formation of interphases between Na and Na_3_Zr_2_Si_2_PO_12_ [168]. XPS findings demonstrated that a kinetically stabilised interface forms upon contact, which resists continuous degradation and supports prolonged cycling. In addition, in-situ impedance spectroscopy was applied to evaluate the electrochemical response of Sc-doped NASICON SSEs under thermal extremes [169]. By tracking impedance evolution during cycling, a strong correlation between Sc substitution, improved bulk and robust interfacial kinetics was established.

In-situ synchrotron-based techniques have also become an effective tool for investigating the electronic structures of Na SSEs, especially towards unveiling the Na-ion transport mechanisms and interfacial dynamics. In a representative study, in-situ micro-X-ray absorption near-edge spectroscopy (μXANES) was used to track the Na|Na_3_Zr_2_Si_2_PO_12_ interfacial evolution under varying electrochemical conditions [170]. By directing a highly focused X-ray beam at the cell and incrementally increasing the applied current density, the study recorded Zr K-edge absorption spectra at high spatial resolution. The results revealed the emergence of a localised interfacial phase, distinct from the Na_3_Zr_2_Si_2_PO_12_ bulk, indicative of Zr^4+^ reduction during cycling. These findings were confirmed through custom-built symmetric Na|Na_3_Zr_2_Si_2_PO_12_|Na cells, designed specifically to capture in-situ interface behaviour (Fig. 14b). Figure 14c–f illustrates the evolution of Zr K-edge XANES spectra and their derivatives for both the Na_3_Zr_2_Si_2_PO_12_ bulk and the interphase formed at the Na|Na_3_Zr_2_Si_2_PO_12_ interface during current-stepped cycling. In bulk Na_3_Zr_2_Si_2_PO_12_ (Fig. 14c,d), the spectra remain almost constant during all cycles, without noticeable edge shift or emergence of pre-edge features. This confirms that the Na_3_Zr_2_Si_2_PO_12_ framework retains its structural integrity, even after extended cycling and short circuits. In contrast, the Na|Na_3_Zr_2_Si_2_PO_12_ interfacial region (Fig. 14e,f) shows a progressive shift of the absorption edge toward lower energies as cycling proceeds, pointing to a gradual reduction of Zr at the interface. According to the spectral results, a chemically modified interphase forms during Na plating, which acts as a passivating layer and prevents subsequent parasitic reaction between Na anode and Na_3_Zr_2_Si_2_PO_12_ [170].

**Fig. 15:**
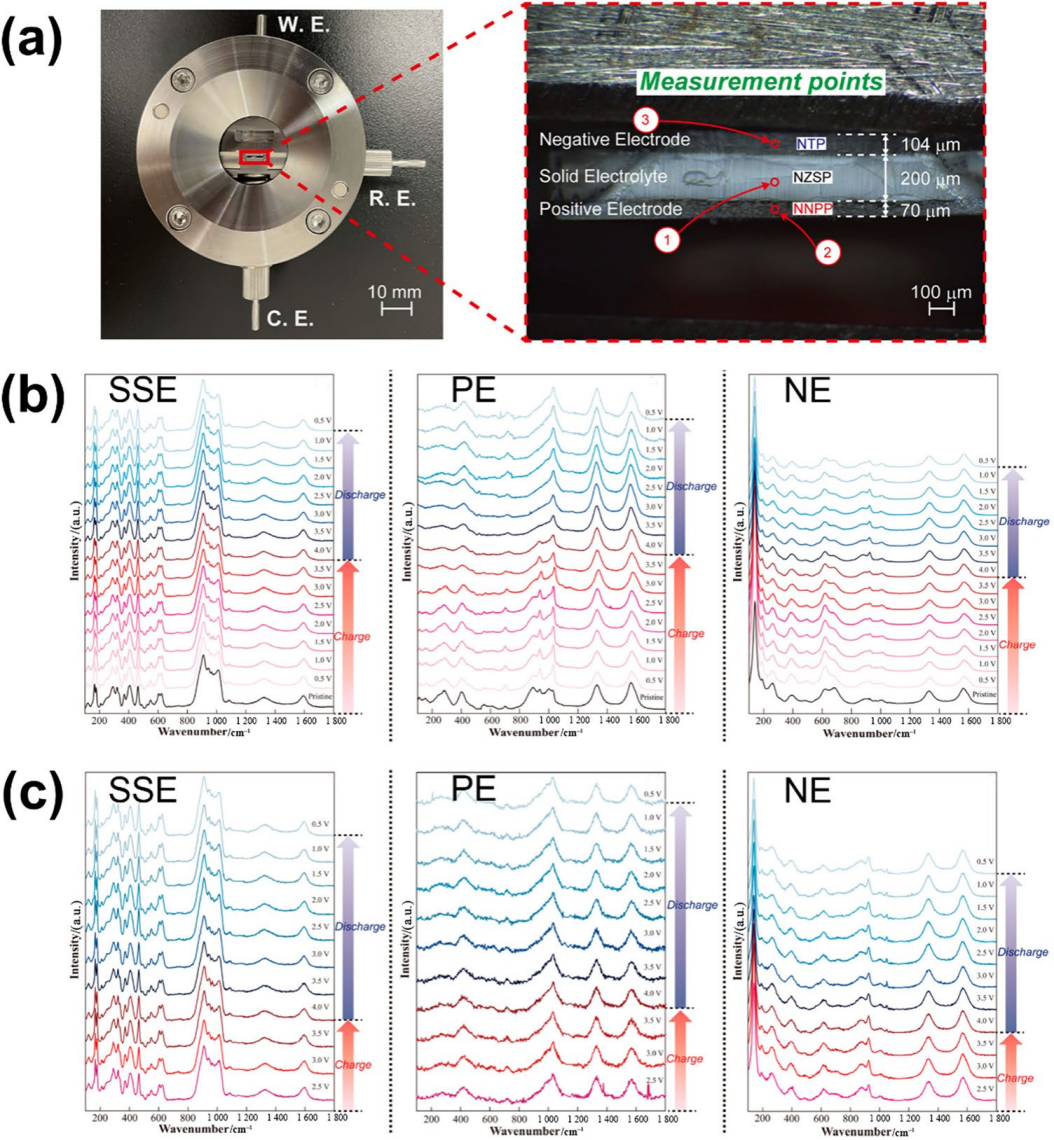
**a** Experimental architecture of ASSNIB for operando Raman test (left panel) and microscopic image showing the measurement points (marked as red circles) across ASSNIB cross-section. Negative electrode (NE): NaTi_2_(PO_4_)_3_ (NTP); SSE: NZSP; positive electrode (PE): Na_4_Ni_3_(PO_4_)_2_P_2_O_7_ (NNPP). Operando Raman spectra of PE, SSE and NE layers at the **b** first and **c** third cycles. Reproduced with permission from Ref. [171]. Copyright © 2023, American Chemical Society

In a representative study, operando Raman spectroscopy was employed to investigate structural changes across a full [NaTi_2_(PO_4_)_3_|Na_3_Zr_2_Si_2_PO_12_|Na_4_Ni_3_(PO_4_)_2_P_2_O_7_] cell [171]. Figure 15a illustrates the experimental configuration for operando Raman measurements used to study the cross-sectional structure of ASSNIBs. The sample was prepared with a flat cross-section via Ar-ion milling, enabling precise operando Raman measurements across the negative electrode, SSE, and positive electrode layers under electrochemical cycling. Red circles mark the measurement points selected for real-time spectral tracking during electrochemical tests. This setup allowed spatially resolved monitoring of chemical and structural changes within the full-cell architecture. Figure 15b and c present operando Raman spectra of ASSNIB during the first (Fig. 15b) and third (Fig. 15c) CV cycles, offering insights into the structural evolution of the SSE (left panel), positive electrode (PE, middle panel), and negative electrode (NE, right panel) layers. The SSE layer exhibited no observable shifts or peak changes throughout cycling, indicating its structural and chemical robustness under electrochemical operation. In contrast, the PE displayed clear spectral changes during the first cycle, particularly around 700 and 800–1 050 cm^−1^, which diminished upon discharge. These changes are attributed to Na^+^ intercalation/deintercalation processes affecting phosphate bonding environments and causing irreversible structural alterations. However, spectra stabilised by the third cycle, suggesting a transition to a metastable configuration following initial capacity loss. Similarly, the NE showed distinct peak variations between 550 and 1 050 cm^−1^ after the first charge, which might stem from the decomposition of metastable species introduced during electrode processing. A sharp peak at ~ 920 cm^−1^ re-emerged after discharge, similar to the original NaTi_2_(PO_4_)_3_ spectrum, implying partial recovery of pristine NaTi_2_(PO_4_)_3_ structure. After three full cycles, both electrodes showed minimal spectral changes, indicating that most structural rearrangements occurred early in cycling. These results help clarify how processing and electrochemical conditions influence electrode degradation mechanisms in ASSNIBs.

Other studies also expand the functional scope of operando analysis, among which operando Raman could also be used to track Na^+^ movement and structural alterations at multiple length scales [172]. Their multi-modal cross-sectional evaluation revealed the compositional gradients and bonding rearrangements at the Na_3_V_2_(PO_4_)_3_|Na_3_Zr_2_Si_2_PO_12_ interface, clarifying how electrochemical reactions unfold across interfaces. Moreover, combining operando SEM–EDS with Raman spectroscopy, spatially resolved insights were provided, showing that Na^+^ migration and bonding rearrangements could be correlated at both micrometre and atomic scales. In another study, operando XPS was utilised to quantify interphase formation kinetics at Na|Na_3_Zr_2_Si_2_PO_12_ interfaces [173]. By tracking the evolution of chemical species during controlled Na plating, the stabilising role of a native Na_*x*_PO_*y*_ surface layer was revealed, which was demonstrated to retard deleterious reactions at the Na|Na_3_Zr_2_Si_2_PO_12_ interface, facilitating the formation of a kinetically stable, electronically insulating interphase. Additionally, operando impedance spectroscopy was applied to explore vacancy-driven pore formation at Na|Na_3_Zr_2_Si_2_PO_12_ interfaces, which demonstrated that incorporating a Na_15_Sn_4_ alloy can improve Na^+^ diffusivity, delay void formation and enhances cycling stability. Operando electrochemical impedance measurements, combined with an analytical diffusion model, revealed that Na_15_Sn_4_ significantly accelerates vacancy annihilation kinetics at the anode, thus mitigating pore formation at the Na|Na_3_Zr_2_Si_2_PO_12_ interface even under high current densities [174].

To summarise, advanced characterisation in-situ and operando techniques have become indispensable for probing the dynamic processes in ASSNIBs, enabling real-time visualisation of morphological, chemical, and electrochemical changes that are often missed in ex-situ investigations. By capturing intermediate states and transient interphases under realistic electrochemical conditions, these methods have significantly advanced the mechanistic understanding of dendrite formation, phase transitions, and interfacial evolution in Na SSE systems. Nevertheless, several limitations remain. For example, the instrumental complexity of in-situ/operando setups often requires specially designed cells or thin-film architectures that may deviate from practical battery configurations. Additionally, the interpretation of data from techniques such as XANES, XPS, and Raman spectroscopy necessitates careful correlation with electrochemical states to avoid misattribution. Besides, in some specific cases, operating conditions may need to be modified, such as exploiting lower current densities or thinner electrodes, to accommodate real-time measurements, potentially limiting the transferability of results to commercial devices. Looking forward, leveraging these real-time advanced characterisation insights to guide rational material optimisation and interface engineering offers a powerful path toward the development of high-performance ASSNIBs. By systematically linking the observed dynamic behaviour with structural features and electrochemical outcomes, better design of SSE compositions, electrode architectures, and cell configurations that can suppress degradation and enhance long-term performance might be achieved.

#### Machine Learning-Accelerated Electrolyte Discovery

**Fig. 16:**
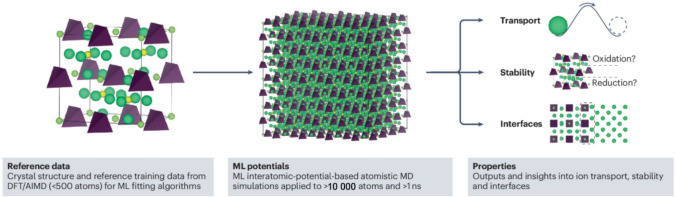
Typical three-step flow charts of machine learning (ML) interatomic-potential-based simulations for SSEs and output properties. Reproduced with permission from Ref. [161]. Copyright © 2025, Springer Nature

Machine learning has recently emerged as a powerful complement to conventional atomistic modelling for Na SSEs, which is poised to further revolutionise electrolyte discovery, enabling accelerated screening, property prediction and mechanistic interpretation across complex chemical spaces [161]. By learning from existing datasets generated through DFT, AIMD, or experimental measurements, machine learning models can predict key descriptors such as formation energy, bandgap, Na-ion diffusivity, and electrochemical stability across broad chemical spaces. Moreover, machine learning models are now being embedded into force fields and atomistic simulations to enable long-timescale modelling of Na-ion transport in disordered systems, which are tricky for traditional modelling methods. The typical workflow (as illustrated in Fig. 16) usually involves three steps: (1) generating high-fidelity training data from first-principles simulations or experimental structures; (2) fitting and validating machine learning potentials to reproduce essential structural and energetic features; (3) applying these potentials to run large-scale MD simulations that yield insights into ionic conduction, material stability, and interface behaviour.

Machine learning-driven force field simulations were used to explore Na^+^ transport behaviour in oxygen-doped NaPSO glassy electrolytes [175]. Traditional molecular dynamics approaches are computationally intensive when applied to disordered systems like glasses. To overcome this, a machine-learning force field was developed using a concurrent learning framework (DP-GEN), which iteratively improves model quality through a cycle of exploration, DFT-based labelling and neural network training. The resulting machine learning force field enabled accurate modelling of Na^+^ diffusion in amorphous matrices over extended time and length scales. The simulations uncovered an unexpected dual role of oxygen doping: although oxygen substitution reduced free volume and tightened the structural network, it simultaneously enhanced amorphous framework flexibility, which paradoxically promoted Na^+^ mobility. This "flexible doorway" diffusion mechanism could not be readily predicted by traditional free-volume theories, showcasing the importance of atomic-scale dynamic analysis in designing high-performance glassy SSEs. Moreover, the machine learning force field enabled tracking of subtle deformation modes and local environment fluctuations, revealing that flexibility in bottleneck regions plays a more critical role than overall free volume for Na^+^ diffusion in glassy electrolytes. Such detailed mechanistic insights provide a rational basis for further optimising dopant concentration and amorphous network topology.

An interpretable machine learning framework known as descriptors-divide-and-conquer machine learning (descriptors-DCML) was applied to predict Na-ion migration barriers in NASICON-type SSEs [176], which combines rough set theory with materials domain knowledge to extract multiple descriptor combinations, enabling a multifaceted view of structure–activity relationships. Different from conventional models applying a single descriptor set, descriptors-DCML divides the problem into several descriptor clusters based on core features, allowing for the construction of multiple high-precision models. This strategy not only leads to the enhancement of accuracy but also reveals hidden mechanistic rules by identifying diverse descriptor sets governing Na-ion transport, thus providing deeper insights compared to the single-descriptor approaches. Among the extracted relationships, in addition to theories, such as the effects of bottleneck size and framework connectivity on ionic conduction, adapting to previous conclusions, some less intuitive factors, including the influence of framework distortion parameters, local Na coordination environments and subtle variations in oxygen polyhedral connectivity are also unveiled and require further experimental validation. Combining the data-driven findings with current studies, descriptors-DCML provides both high predictive accuracy of 93.8% and strong physical interpretability, showing the strong power of machine learning in discovering material design principles.

In summary, machine learning has become a valuable enabler in the development of Na SSEs, supporting both predictive modelling and accelerated screening of candidate materials. By integrating with computational methods such as DFT and molecular dynamics, machine learning models allow for efficient exploration of complex material spaces and uncover hidden structure–property relationships that are difficult to access using traditional approaches. These capabilities help reduce the time, cost, and effort required for electrolyte discovery and optimisation. However, several challenges remain, such as the requirement for large, high-quality, and diverse datasets to ensure predictive accuracy. Besides, the interpretability of complex models like deep neural networks can be limited, making it difficult to extract intuitive physical insights. Moreover, generalising these models to novel material systems still requires careful validation, both by theoretical verification and experimental results. Looking forward, the integration of machine learning with growing high-throughput experimental and computational datasets, including structural and electrochemical libraries, may enable automated diagnostics of interfacial structures, defect landscapes, and reaction pathways, thereby supporting rational design strategies for high-performance Na SSE materials and corresponding ASSNIBs.

The information provided, strengths and limitations of the advanced characterisation and modelling techniques described above are summarised in Table 3.

**Table 3:** Summary of the capabilities, strengths and limitations of advanced characterisation and modelling techniques in the studies of Na SSEs

Techniques	Capabilities	Strengths	Limitations	References
Cryo-TEM/Cryo-STEM	Visualising the structural evolution of electrodes, Na SSEs and their interphases at the atomic to nanoscale; Mapping of elemental distributions across the devices when coupled with EDS	Enables direct, high-resolution imaging of electrodes, Na SSEs and their interphases; Minimises beam-induced artifacts, preserving the original structure of materials; Reveals crystalline/amorphous features at the nanoscale	Requires complex sample preparation under cryogenic conditions; Limited field of view and sampling volume; Complicated structural interpretation due to overlapping signals	[162, 163]
In-situ/Operando techniques	Monitoring dendritic growth dynamics; Tracking the whole process of interphase formation and evolution; Understanding ion transport and structural evolution at the interfaces	Real-time tracking of chemical, structural, and morphological changes during battery operation; Captures dynamic processes under realistic electrochemical conditions; Applicable across multiple scales (atomic to macroscopic)	Instrumental complexity Data interpretation requires careful correlation with electrochemical states Sometimes the testing conditions need to be modified to fit to experiments	[164–174]
Machine learning	Virtual screening of Na SSE composition; Prediction of Na-ion conducting behaviour and stability windows; Mechanistic insights into Na-ion transport behaviour in Na SSEs	Enables high-throughput screening of material candidates; Predicts physical, chemical and electrochemical properties with DFT-level accuracy at lower cost; Uncovers complex structure–property relationships and hidden Na-ion transport mechanisms	Requires large, high-quality datasets for model training; Interpretability of the rules obtained from deep learning models can be limited; Generalisation to unknown material systems still needs careful validation	[161, 175–177]

## Improvement Strategies in Na SSEs

**Fig. 17:**
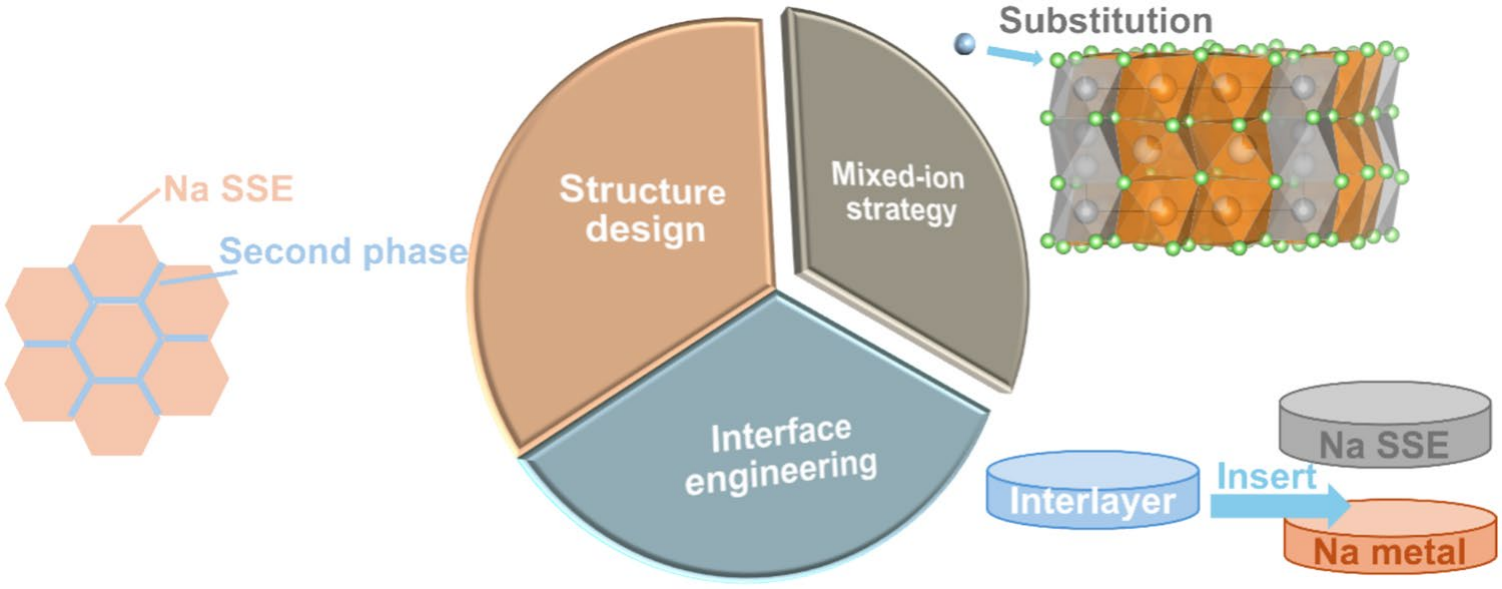
Three improvement strategies for Na SSEs

Based on the understanding of Na-ion conduction mechanism and interfacial evolution process obtained from the advanced characterisation and modelling techniques, research efforts aimed at enhancing Na SSEs and corresponding ASSNIBs have gained momentum to address current issues in Na SSEs. Figure 17 shows the three key strategies to improving the performance associated with Na SSEs, including microstructural design, mix-ion strategy, and interface engineering.

### Microstructural design

**Fig. 18:**
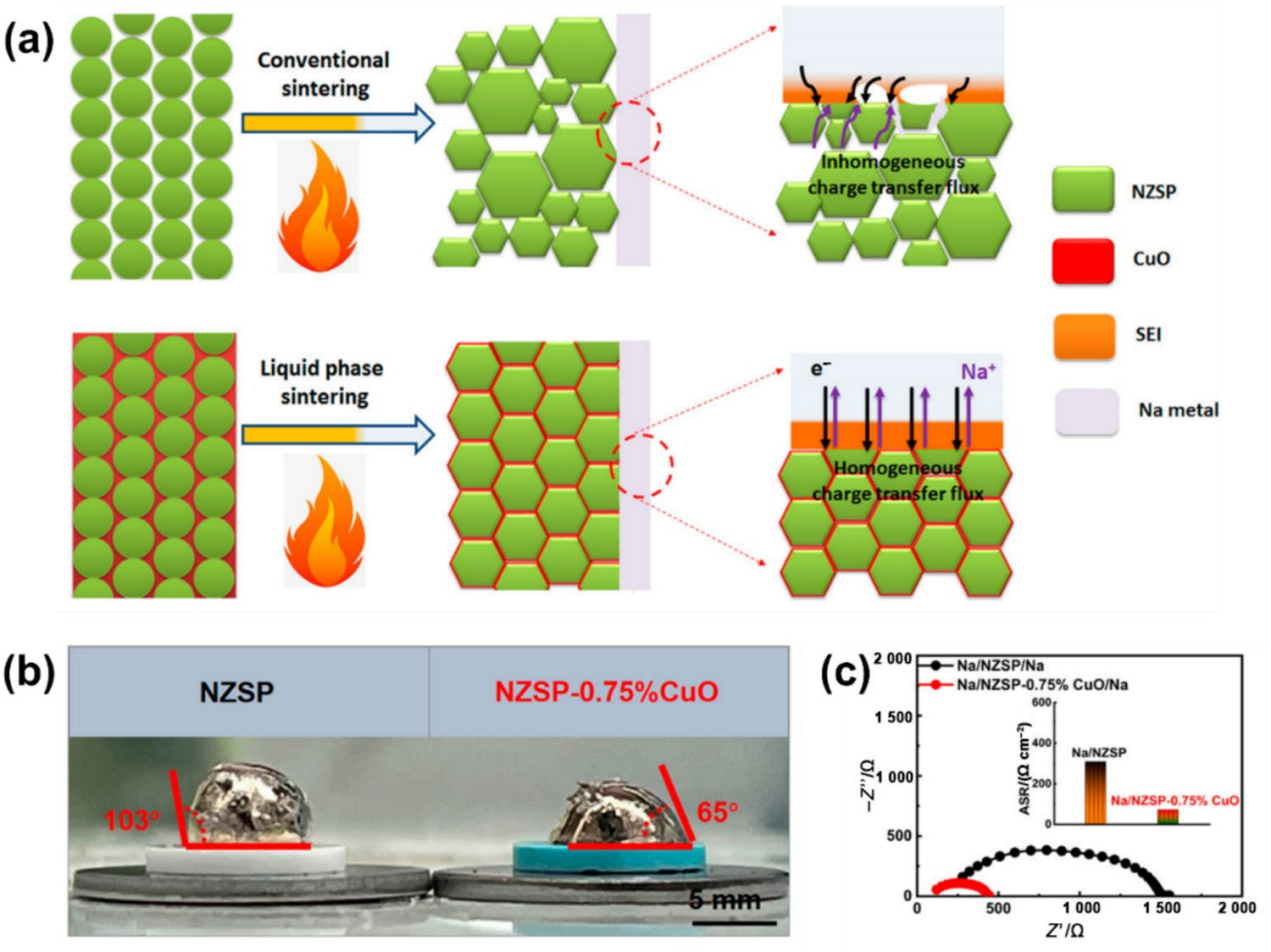
**a** Schematic illustration of preparing Na_3_Zr_2_Si_2_PO_12_ with/without CuO, which optimises the microstructure of SSE and facilitates the formation of stable solid electrolyte interphase (SEI). **b** Optical images illustrating the wettability between Na/Na_3_Zr_2_Si_2_PO_12_ and Na_3_Zr_2_Si_2_PO_12_-0.75%CuO at 150 °C. **c** Nyquist plots of Na symmetric cells using Na_3_Zr_2_Si_2_PO_12_ and Na_3_Zr_2_Si_2_PO_12_-0.75%CuO as SSEs. (inset: area specific resistance of Na/Na_3_Zr_2_Si_2_PO_12_ and Na_3_Zr_2_Si_2_PO_12_-0.75%CuO interface). Reproduced with permission from Ref. [180]. Copyright © 2022, American Chemical Society

To optimise the mechanical properties of Na SSEs and effectively suppress Na dendrite propagation in ASSNIBs, modulating the microstructure of Na SSEs has been demonstrated as a viable strategy. In a representative study, nanoindentation measurements revealed that the hardness of NASICON-type Na_1+*x*_Mn_*x*/2_Zr_2−*x*/2_(PO_4_)_3_ decreased from 7.8 GPa to 4.2 GPa with the increased Na content from *x* = 0.5 to 2.0, indicating the gradual softening of NASICON SSE with higher Na concentration [178], and thin Al-doped Li_7_La_3_Zr_2_O_12_ garnet electrolyte exhibited higher elastic modulus (~ 155 GPa), hardness (~ 11 GPa), and nanoindentation fracture toughness (~ (1.12 ± 0.12) MPa m^1/2^) than the bulk sample (~ 142 GPa, ~ 10 GPa, and ~ (0.97 ± 0.1) MPa m^1/2^, respectively) [179]. These mechanical trends suggest that careful modulation of SSE structure will enhance its mechanical strength to resist dendritic penetration. Therefore, integrating a secondary phase into Na SSEs, which tunes the SSE structure while maintaining its originally excellent electrochemical properties, was exploited in many studies. A good example is the incorporation of CuO as an intergranular phase in NASICON Na_3_Zr_2_Si_2_PO_12_, which was demonstrated to play a crucial role in mitigating Na dendrite formation and enhancing battery performance [180]. Conventional sintering of Na_3_Zr_2_Si_2_PO_12_ often results in uneven grain structures, which can lead to localised charge accumulation and promote dendritic growth (Fig. 18a). In contrast, the introduction of CuO can influence the sintering process by facilitating a liquid-phase mechanism, leading to more compact and uniformly distributed grains. This structural refinement not only minimises grain boundary resistance but also ensures a more consistent distribution of Na-ion transport pathways, reducing the risk of concentrated ion flux that could trigger dendrite formation. A key aspect of the effectiveness of CuO in stabilising the Na interface is the formation of a conductive Na − Cu − O layer. This interphase, which emerges due to interfacial reactions, improves charge transport efficiency while preventing direct contact between Na and Na_3_Zr_2_Si_2_PO_12_, thereby reducing the likelihood of dendrite penetration. Improved Na wettability is another benefit, as demonstrated by the reduction in contact angle from 103° to 65° (Fig. 18b), which facilitates more stable Na deposition and lowers interfacial resistance. The impact of this modification is evident in EIS data, where the resistance in Na symmetric cells using CuO-modified Na_3_Zr_2_Si_2_PO_12_ drops significantly, from 1 500 to 500 Ω (Fig. 18c). Additionally, the area-specific interfacial resistance (ASR) decreases from 308 to 70 Ω cm^2^, signifying enhanced electrode–electrolyte compatibility. Beyond these microstructural and interfacial improvements, CuO modification has a profound impact on device performance. Na symmetric cells utilizing the modified electrolyte exhibit exceptional cycling stability, even at current densities reaching 0.4 mA cm^−2^, whereas unmodified Na_3_Zr_2_Si_2_PO_12_ cells fail prematurely due to dendrite-induced short-circuiting. This significant improvement underscores the role of CuO in enabling stable long-term operation, making it a promising approach for high-performance ASSNIBs.

In addition, Na_3_Zr_2_Si_2_PO_12_ integrated with ferroelectric BaTiO_3_ phase or TiO_2_ phase [181, 182] also shows compact morphology and enhanced mechanical properties further enhancing its ability to inhibit Na dendrite growth and achieve excellent electrochemical performance.

### Mixed-ion strategy

Na-ion transport in SSEs is a complicated process influenced by various factors, including defects, crystal structures, channel sizes and crystallinity [27, 183–185]. The ionic conductivity of SSEs can be adjusted based on two primary aspects, charge carrier concentration (*n*) and charge carrier mobility (*μ*), as described by the equation:

$$\sigma =qn\mu$$

where *q* represents charge quantity of the carrier. As Na^+^ is the only research object in this system, so the value of *q* is fixed.

To modulate charge carrier concentration and mobility, mixed-ion strategy, also known as heteroatomic doping, has been demonstrated as effective. Doping with different ions can alter both the Na-ion concentration and the size of the diffusion channels [111, 186]. This approach can be categorised into two types of SSEs: mixed-cation and mixed-anion compounds.

#### Mixed-cation compounds

**Fig. 19:**
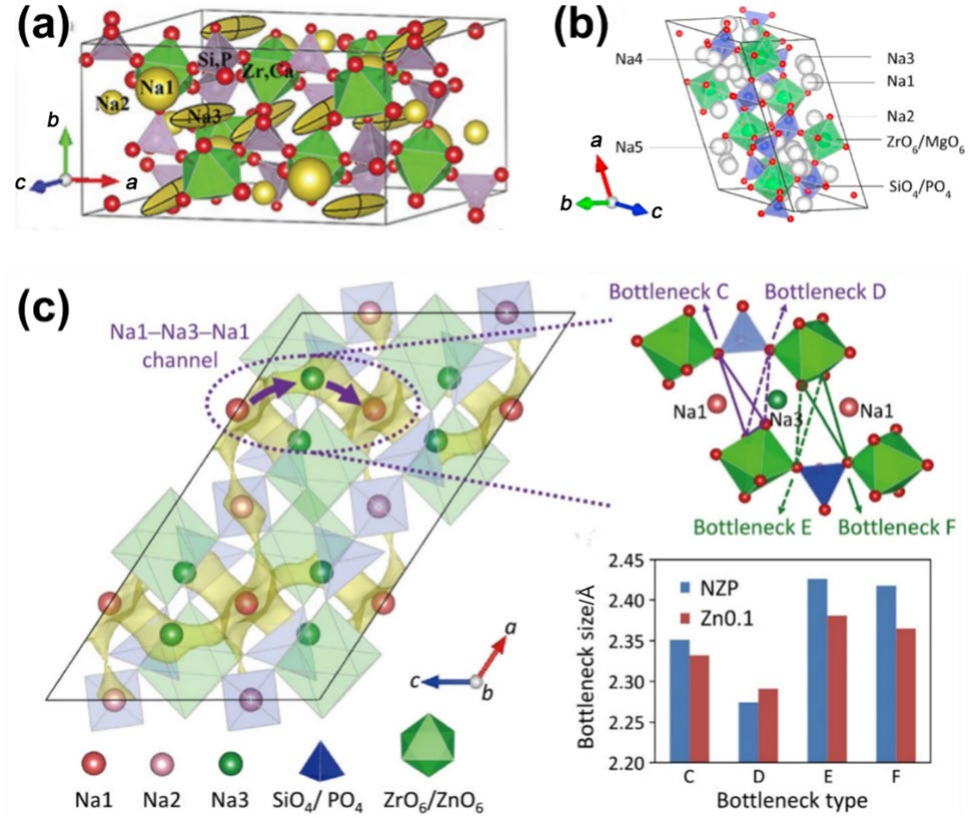
**a** Crystal structure of Ca-doped NASICON. Reproduced with permission from Ref. [195]. Copyright © 2019, Wiley–VCH and **b** Mg-doped NASICON. Reproduced with permission from Ref. [196]. Copyright © 2021, Elsevier. **c** BVEL map of Na_3.2+2*x*_Zr_2−*x*_Zn_*x*_Si_2.2_P_0.8_O_12_, in which purple arrows indicate Na1 − Na3 − Na1 ion-migration pathways. The corresponding bottlenecks are displayed in the upper right panel, and the sizes of bottlenecks of Na_3.2_Zr_2_Si_2.2_P_0.8_O_12_, and Na_3.4_Zr_1.9_Zn_0.1_Si_2.2_P_0.8_O_12_ are shown in the lower right panel. Reproduced with permission from Ref. [197]. Copyright © 2020, American Chemical Society

To enhance the performance of Na oxide SSEs, mixed-cation compounds have been developed. In addition to dopants such as Ti [187–189], Co [190], Mg [191, 192], Ni [193] and Y [194] in *β*-alumina SSEs, research has also focused on NASICON-type SSEs, leading to the synthesis of a series of superionic conductors. For instance, calcium-doped NASICON, synthesized via sol–gel preparation, exhibits a high ionic conductivity of 1.67 mS cm^−1^ [195]. Figure 19a displays the structure of Na_3.4_Zr_1.8_Ca_0.2_Si_2_PO_12_, which features a monoclinic structure with *C*2/*c* symmetry. The framework is composed of (Zr, Ca)O_6_ octahedra and (P, Si)O_4_ tetrahedra that share corners, forming a three-dimensional network for Na^+^ migration. There are three types of Na sites within the ion-diffusion channels formed by these units, with the [Na3–Na1–Na3] pathway identified as the most favourable for Na^+^ migration. The enhanced ionic conductivity is attributed to the improved Na^+^ mobility at the Na_*3*_ sites following calcium substitution.

Similarly, Mg^2+^ substitution in NASICON yielded an impressive ionic conductivity of 3.6 mS cm^−1^ in Na_3.4_Mg_0.1_Zr_1.9_Si_2.2_P_0.8_O_12_ [196]. Figure 19b depicts its monoclinic structure, where [Zr, Mg]O_6_ octahedra and [P, Si]O_4_ tetrahedra also share corners, forming Na-ion transport pathways akin to those in Ca-doped NASICON. The increased lattice size following Mg substitution contributes to the enhanced ion conductivity by providing more space for Na^+^ transport. Additionally, Zn^2+^-substituted NASICON Na_3.4_Zr_1.9_Zn_0.1_Si_2.2_P_0.8_O_12_ exhibits a remarkable RT ionic conductivity of 5.27 mS cm^−1^ [197]. BVEL analysis indicates that the enlarged bottlenecks in the Na-ion transport pathways account for this increased ionic conductivity (Fig. 19c). In [Na1 − Na3 − Na1] pathway, four oxygen-triangle bottlenecks (C, D, E, F) are observed. After Zn doping the size of bottleneck D increased from 2.274 to 2.291 Å, facilitating fast Na^+^ migration. The higher Na^+^ content further contributes to the enhanced ionic conductivity.

**Fig. 20:**
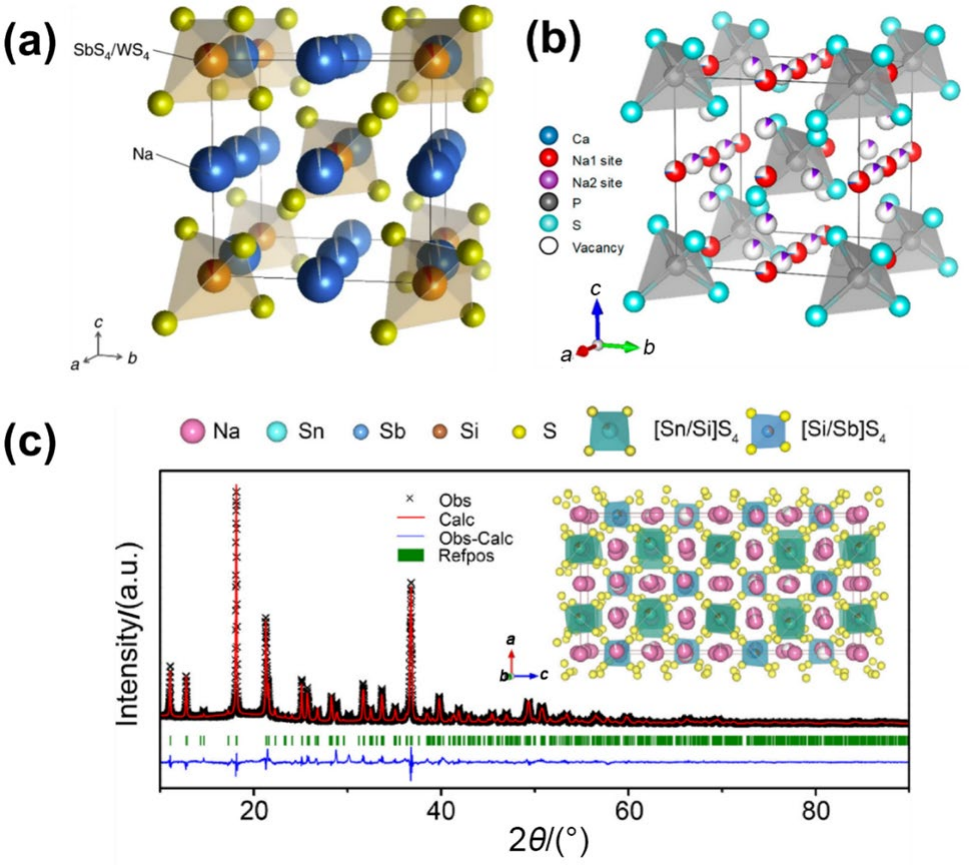
Crystal structure of** a** W-substituted Na_3_SbS_4_ [200]. **b** Ca-substituted Na_3_PS_4_ Reproduced with permission from Ref. [211]. Copyright © 2018, American Chemical Society. Rietveld refinement and corresponding crystal structure of **c** Sb-substituted Na_4_Sn_0.67_Si_0.33_S_4_. Reproduced with permission from Ref. [207]. Copyright © 2020, Elsevier

Similar to Na oxide SSEs, the mixed-cation strategy has been applied to both Na_3_PS_4_-type and Na_11_SnP_2_S_12_-type SSEs, resulting in the development of numerous mixed-cation Na sulfide SSEs. These include Ge [198], W [76, 199–202], Ti, Sn [203], Si [204] and As [205, 206] doped Na_3_*Pn*S_4_ (*Pn* = P or Sb), along with varying Na_11_SnP_2_S_12_-type composition such as Na_3.8_[Sn_0.67_Si_0.33_]_0.8_Sb_0.2_S_4_ [207], Na_3.67_[Sn_0.67_Si_0.33_]_0.67_P_0.33_S_4_ [208], Na_11.5_Sn_2_Sb_0.5_Ti_0.5_S_12_ [209] and Na_11+*x*_Sn_2+*x*_(Sb_1−*y*_P_*y*_)_1−*x*_S_12_ [210]. A notable development is the superionic conductor Na_2.88_Sb_0.88_W_0.12_S_4_, formed by substituting partial Sb with W, which exhibits an impressive RT ionic conductivity of 32 mS cm^−1^ (Fig. 20a) [200]. This exceptional conductivity is attributed to an increased Na vacancy concentration and the stable cubic phase induced by tungsten substitution. Furthermore, Na_2.88_Sb_0.88_W_0.12_S_4_ demonstrates specific moisture tolerance and enhanced densification at low sintering temperatures.

Calcium substitution in the Na sites of t-Na_3_PS_4_ was also explored, leading to a phase transition from tetragonal to cubic (Fig. 20b) [211]. The synthesized Na_3−2*x*_Ca_*x*_PS_4_ displays a high RT ionic conductivity of approximately 1 mS cm^−1^ when *x* = 0.135, attributed to the increased Na vacancies from calcium doping. Notably, all elements in this compound are earth-abundant and environmentally friendly, making them suitable for large-scale production. Using Na_4_Sn_0.67_Si_0.33_S_4_ as a template compound, Sb-substitution was investigated, achieving the highest ionic conductivity (0.175 mS cm^−1^) in Na_3.8_[Sn_0.67_Si_0.33_]_0.8_Sb_0.2_S_4_ [207]. Rietveld refinement results indicate that Sb-substituted Na_4_Sn_0.67_Si_0.33_S_4_ retains the *I*4_1_/*acd* symmetry of its template, while the enlarged lattice contributes to the enhanced ionic conductivity (Fig. 15c).

**Fig. 21:**
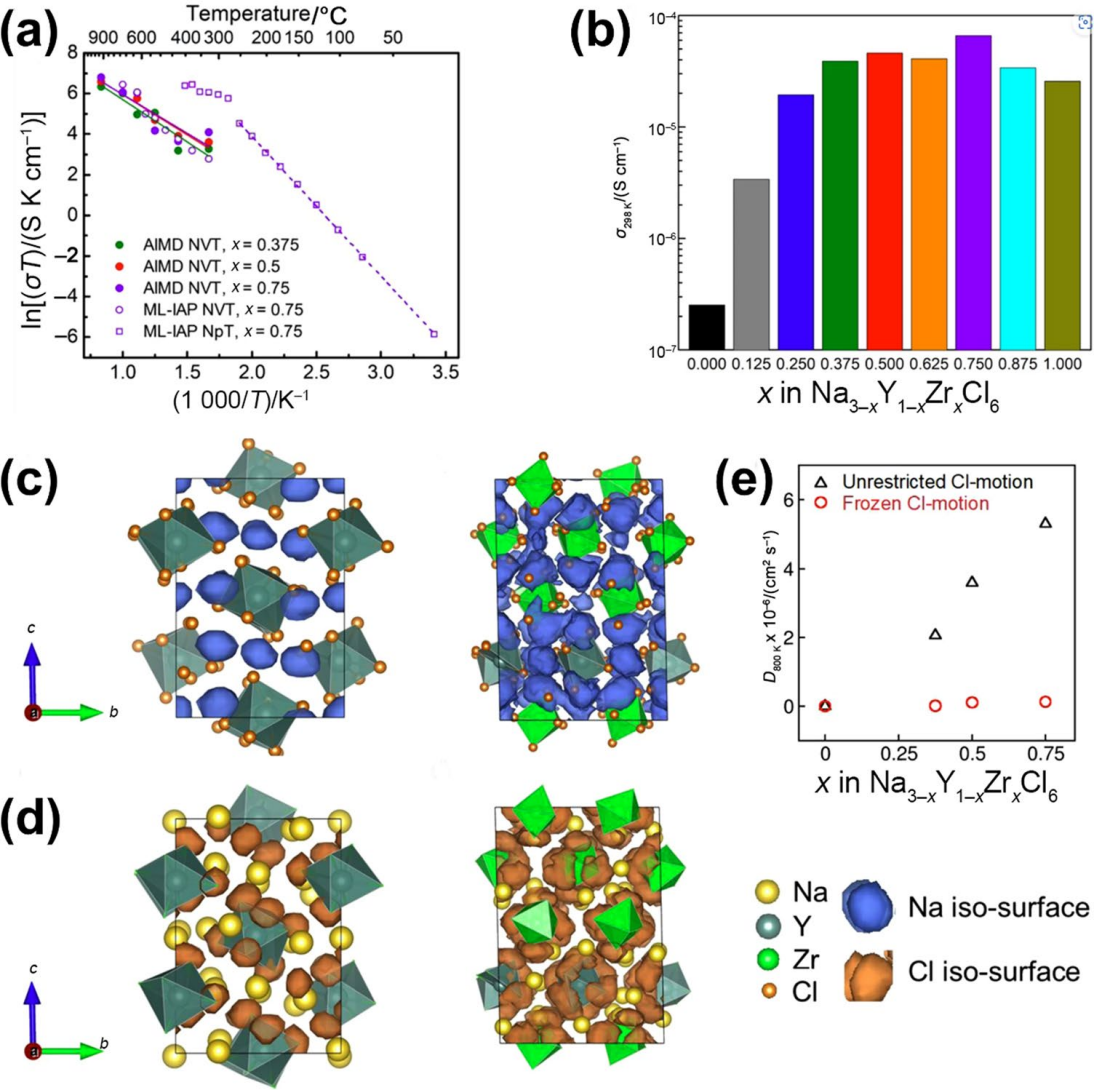
**a** Arrhenius plots of Zr-doped Na_3_YCl_6_ (Na_3−*x*_Y_1−*x*_Zr_*x*_Cl_6_) calculated by AIMD simulations and ML-IAP MD simulations. **b** RT ionic conductivities of Na_3−*x*_Y_1−*x*_Zr_*x*_Cl_6_. Probability density of **c** Na^+^ in Na_3_YCl_6_ (left panel) and Na_2.25_Y_0.25_Zr_0.75_Cl_6_ (right panel) [36], **d** Cl^−^ in Na_3_YCl_6_ (left panel) and Na_2.25_Y_0.25_Zr_0.75_Cl_6_ (right panel) by AIMD simulations [36]. **e** Diffusivity of Na-ion at 800 K for Na_3−*x*_Y_1−*x*_Zr_*x*_Cl_6_, with Cl^−^ in either frozen or unrestricted space [36]

Like the Li-based counterparts [212–216], the mixed-cation strategy has also been applied to modify Na halide SSEs. The first reported mixed-cation Na halide SSE, Na_3−*x*_Er_1−*x*_Zr_*x*_Cl_6_ shows an increase in ionic conductivity from 10^−6^ mS cm^−1^ to 0.035 mS cm^−1^ when *x* = 0.6 [217]. This enhancement is attributed to the replacement of Er^3+^ with small-radius Zr^4+^, which rearranges the polyhedral motifs and increases Na^+^ vacancy concentration. Unlike Li_3_YCl_6_, Na_3_YCl_6_ exhibits a significantly lower ionic conductivity due to the absence of partial Na-site occupancy at the *2d* and *4e* positions. To enhance its ionic conductivity, Zr^4+^ substitution was introduced, leveraging its low dopant formation energy and cost-effectiveness [36]. AIMD simulations predicted negligible Na^+^ diffusion in pristine Na_3_YCl_6_, whereas Zr^4+^-substituted compositions exhibited markedly improved diffusion behaviour (Fig. 21a). To further investigate ion transport at lower temperatures, a machine learning interatomic potential (ML-IAP) based on the moment tensor potential (MTP) formalism was developed, trained using AIMD snapshots and structural data. The simulations revealed a transition between two linear regimes in the Arrhenius plot near 500–550 K. Below this temperature range, an activation energy of 594 meV was calculated, and the RT ionic conductivity was estimated at 0.014 mS cm^−1^, two orders of magnitude higher than that of pristine Na_3_YCl_6_. Experimental validation confirmed the improved ionic conductivity in Zr-substituted compositions. The Na_3−*x*_Y_1−*x*_Zr_*x*_Cl_6_ series (0.375 ⩽ *x* ⩽ 1) exhibited conductivities ranging from 0.026 to 0.066 mS cm^−1^, with the highest value (0.066 mS cm^−1^) observed for Na_2.25_Y_0.25_Zr_0.75_Cl_6_ (Fig. 21b) [36]. At higher Zr concentrations (*x* ⩾ 0.875), conductivity declined, likely due to the formation of the poorly conducting crystalline Na_2_ZrCl_6_ phase. To understand the mechanism behind enhanced conductivity, probability distributions of Na-ion motion at elevated temperatures were obtained from AIMD trajectories. In Na_3_YCl_6_, Na^+^ ions primarily exhibit localized transport (Fig. 21c, left panel), whereas fast, long-range 3D motion is observed in Na_2.25_Y_0.25_Zr_0.75_Cl_6_ (Fig. 21c, right panel) [36]. Similarly, the anion dynamics differ, where Cl^−^ ions remain largely static in Na_3_YCl_6_, but show pronounced motion in the Zr-substituted compound, indicating active rotation of [YCl_6_]^3−^ and [ZrCl_6_]^2−^ octahedra (Fig. 21d). To probe the effect of these octahedral rotations, two *gedanken* experiments were performed using AIMD simulations with either frozen or free Cl^−^ motion in Na_3−*x*_Y_1−*x*_Zr_*x*_Cl_6_ (Fig. 21e). When anion rotation was suppressed, the Na^+^ diffusion behaviour of the Zr-substituted system resembled that of undoped Na_3_YCl_6_, despite increased Na-vacancy concentrations and cell volume. These results indicate that the enhanced ionic conductivity originates from the effect of increased lattice volume and dynamic octahedral rotations. In another study, the formation of nanocrystalline and amorphous phases in Na_3−*x*_Y_1−*x*_Zr_*x*_Cl_6_, along with preferred Na^+^ occupancy in the local microenvironment, has been shown to facilitate Na^+^ hopping [218].

**Fig. 22:**
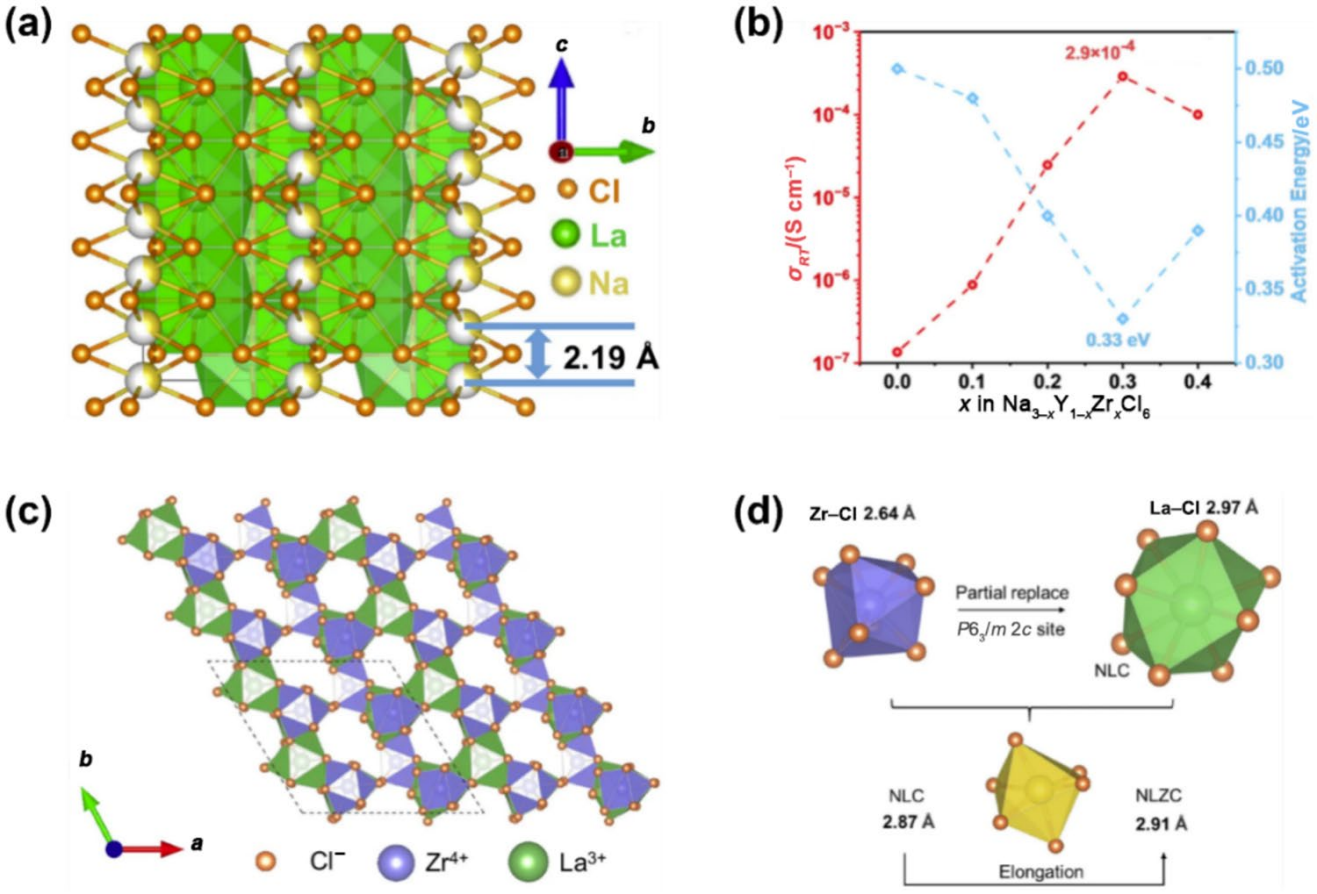
**a** Crystal structure of the NaLaCl_4_, in which white balls represent vacancies. **b** Ionic conductivities and activation energies of Na_1−*x*_Zr_*x*_La_1−*x*_Cl_4_ (0 ⩽ *x* ⩽ 0.4). **c** Supercell of Na_1−*x*_Zr_*x*_La_1−*x*_Cl_4_ in *ab* plane. **d** Bond lengths of Cl with La, Zr, and Na in NaLaCl_4_ or Na_1−*x*_Zr_*x*_La_1−*x*_Cl_4_ [221]

With promising advances in UCl_3_-based Li halide SSEs achieving ionic conductivities of up to 1 mS cm^−1^ [219, 220], a similar structure has been explored in Na-based systems [221]. The structure of NaLaCl_4_ is depicted in Fig. 22a, where Na^+^ occupies partial octahedral sites with adjacent-site distance of 2.19 Å, enabling fast Na^+^ transport. By introducing Zr^4+^ into NaLaCl_4_, the highly conductive Na_1−*x*_Zr_*x*_La_1−*x*_Cl_4_ was obtained, exhibiting an ionic conductivity of 0.29 mS cm^−1^ and a low activation energy of 0.33 eV when *x* = 0.3 (Fig. 22b). Theoretical simulations were also employed to identify the optimal structure and the mechanism underlying the enhanced ionic conductivity in Na_1−*x*_Zr_*x*_La_1−*x*_Cl_4_. The favourable structure, as shown in Fig. 22c, indicates that the substitution of Zr^4+^ at previous La^3+^ sites results in shorter M–Cl lengths (2.64 Å for Zr–Cl and 2.97 Å for La–Cl) owing to the higher oxidation state and smaller radius of Zr^4+^ (Fig. 22d). Conversely, the Na–Cl length increases from 2.87 Å to 2.91 Å, widening the bottleneck in the 1D ion-diffusion channel and lowering the site energy, ultimately enhancing ionic conductivity.

#### Mixed-Anion Compounds

In addition to mixed-cation SSEs, mixed-anion compounds have also been explored, which can be classified into oxysulfide, oxyhalide, and sulfur halide SSEs based on their anion components.

Mixed oxygen-sulfur materials, derived from Na_3_PS_4_ SSE, have varying O-substitution levels of 0 < *x* ⩽ 0.6 in Na_3_PS_4−*x*_O_*x*_ [222]. The increased concentrations of oxide and oxysulfide units in Na_3_PS_4−*x*_O_*x*_ result in stronger and denser glass networks compared to non-O-doped variants, leading to enhanced mechanical properties. Additionally, the formation of bridging oxygen units promotes pressure-induced sintering, yielding a homogeneous glass microstructure. Similar to oxysulfide materials, Na_3_PS_4−*x*_Se_*x*_ has been investigated, revealing that a tetragonal-to-cubic phase transition was observed with increasing *x* [223]. Studies on Na_3_PS_4−*x*_Se_*x*_ suggest that the multiple local symmetries and polarizability of the anion framework significantly affect Na^+^ transport and the ionic conductivity of the materials [224, 225].

**Fig. 23:**
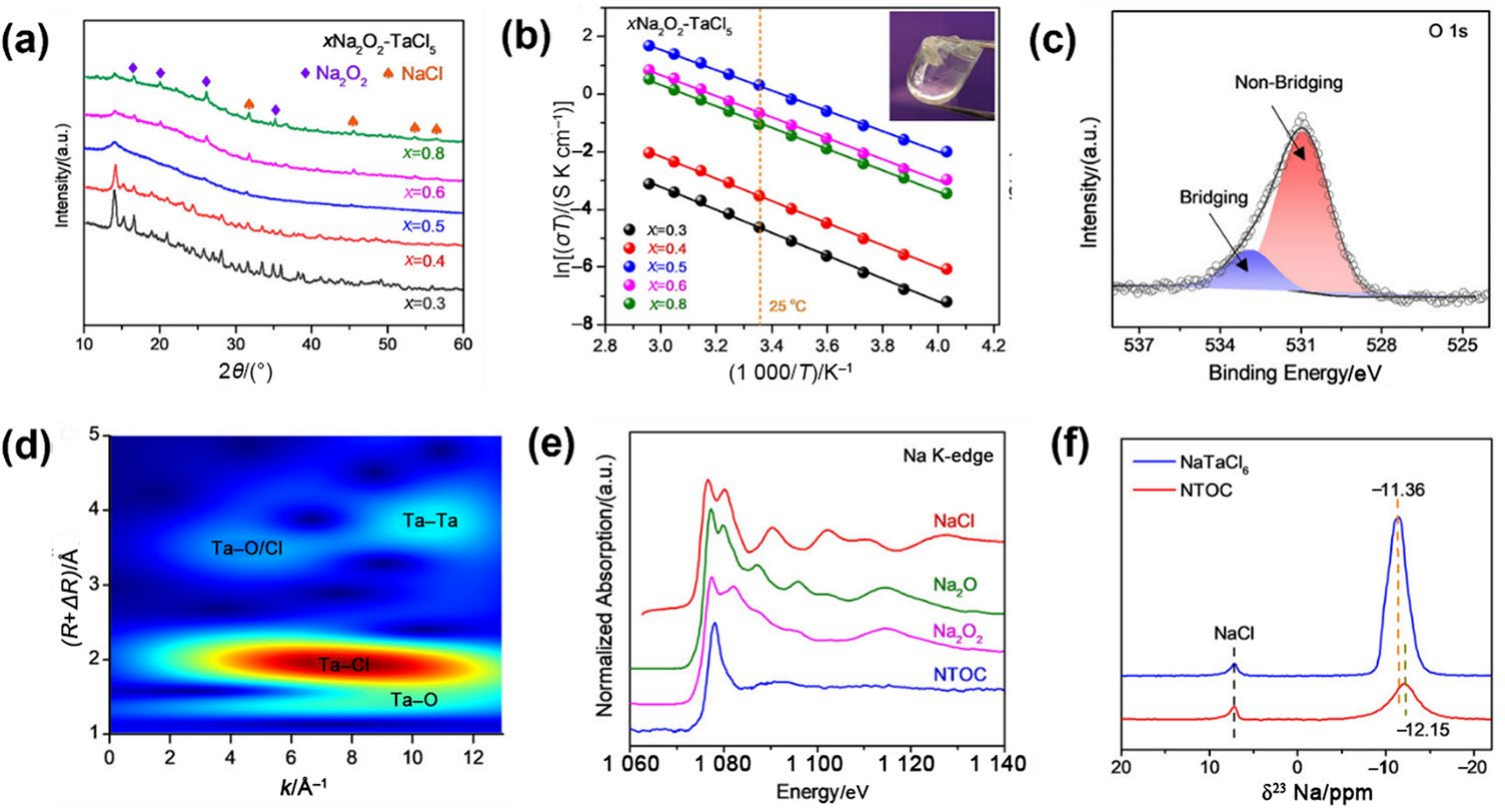
**a** XRD patterns of *x*Na_2_O_2_-TaCl_5_ and **b** their Arrhenius plots (inset: pure 0.5Na_2_O_2_-TaCl_5_). **c** O 1 s XPS spectrum of 0.5Na_2_O_2_-TaCl_5_. **d** WT-EXAFS of 0.5Na_2_O_2_-TaCl_5_ at Ta L_3_-edge. **e** Na K-edge X-ray absorption near-edge spectroscopy (XANES) spectra of 0.5Na_2_O_2_-TaCl_5_, NaCl, Na_2_O and Na_2_O_2_. **f** ^23^Na solid-state NMR spectra of 0.5Na_2_O_2_-TaCl_5_ and NaTaCl_6_. Reproduced with permission from Ref. [160]. Copyright © 2023, Wiley–VCH

Building on successful improvements in ionic conductivity for Li-based halide SSEs [226, 227], oxyhalide Na SSEs have also been explored, utilizing available elements like Ta, Zr, and Al. For example, *x*Na_2_O_2_-TaCl_5_, synthesized by co-melting Na_2_O_2_ and TaCl_5_, demonstrates rapid Na^+^ transport [160]. As shown in Fig. 23a, a pure phase of *x*Na_2_O_2_-TaCl_5_ is observed when *x* = 0.5, while an unknown impurity phase appears for *x* < 0.5 and NaCl is present when *x* > 0.5. With the pure amorphous structure of 0.5Na_2_O_2_-TaCl_5_, the optimal Na^+^ conductivity at *x* = 0.5 reaches 4.62 mS cm^−1^, with a relatively low activation energy of 0.30 eV (Fig. 23b). To understand the rapid Na^+^ transport in this microenvironment, several characterisation techniques were employed. O 1 s XPS revealed two types of oxygen: bridging and non-bridging (Fig. 23c). WT-EXAFS indicated the presence of O and Cl around Ta, suggesting that [TaO_*x*_Cl_*y*_] components are connected through shared O in 0.5Na_2_O_2_-TaCl_5_ (Fig. 23d). Additionally, Na K-edge XAS exhibited specific Na absorption features in 0.5Na_2_O_2_-TaCl_5_, including an intense peak at 1 078 eV and a smooth resonance at 1091 eV, corresponding to the highly disordered Na^+^ environment shown in XRD patterns (Fig. 23e). The ^23^Na solid-state NMR spectrum supported these findings, displaying a wide featureless resonance at 12.15 ppm in 0.5Na_2_O_2_-TaCl_5_ (Fig. 23f). Overall, the structural analysis indicates that the disordered Na^+^ environment and the presence of non-bridging oxygen facilitate the formation of an open anion framework, providing Na^+^ hopping sites critical for fast Na^+^ transport in 0.5Na_2_O_2_-TaCl_5_. Furthermore, the O-substitution strategy was introduced into cost-effective NaAlO_4_ and Na_2_ZrCl_6_, achieving improved ionic conductivity on an order of 0.1 mS cm^−1^ [228–230]. In both materials, the enhanced Na^+^ migration is attributed to the amorphous matrix, similar to the findings in 0.5Na_2_O_2_-TaCl_5_.

Sulfur halide SSEs have also been developed, primarily based on Na_3_PS_4_-type SSEs, with Na_3−*x*_PS_4−*x*_Cl_*x*_ and Na_3−*x*_SbS_4−*x*_Cl_*x*_ serving as notable examples [231–234]. These studies demonstrate that by substituting a small amount of S with Cl, the ionic conductivity of Na_3_PS_4_ or Na_3_SbS_4_ can be significantly enhanced, reaching values that even exceed 1 mS cm^−1^.

### Interface Engineering

To enhance interfacial compatibility between SSEs and electrodes, it is essential to consider both chemical compatibility and interfacial contact, thereby minimising the parasitic reactions and interfacial resistance [235–238].

**Fig. 24:**
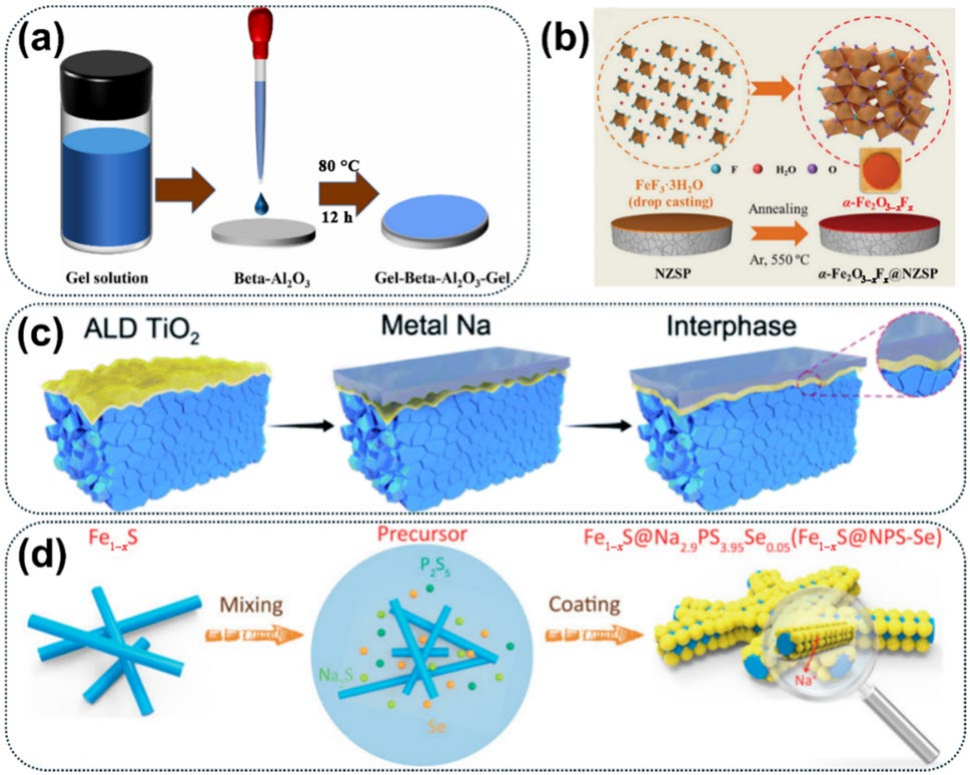
Schematic illustrations of interfacial engineering methods for Na SSEs. **a** Preparation of PVDF-HFP/PMMA layer on *β*-Al_2_O_3_ SSE. Reproduced with permission from Ref. [240]. Copyright © 2022, Elsevier. **b** Preparation of *α*-Fe_2_O_3−*x*_F_*x*_@Na_3_Zr_2_Si_2_PO_12_. Reproduced with permission from Ref. [241]. Copyright © 2024, Wiley–VCH. **c** Preparation of Na|TiO_2_|Na_3_Zr_2_Si_2_PO_12_ and interphase formation. Reproduced with permission from Ref. [244]. Copyright © 2020, Royal Society of Chemistry. **d** Fabrication of Fe_1−*x*_S@Na_2.9_PS_3.95_Se_0.05_ nanorods. Reproduced with permission from Ref. [253]. Copyright © 2018, American Chemical Society

Inserting a buffer layer at the electrode/SSE interface is a plausible strategy that has garnered considerable attention [140, 239], particularly for modifying the anode/SSE interface. For example, coating a poly(vinylidene fluoride-co-hexafluoropropylene)/poly(methyl methacrylate) (PVDF-HFP/PMMA) membrane on *β*-Al_2_O_3_ SSE improves both physical contact and Na-ion migration, reducing interfacial resistance and effectively inhibiting dendritic propagation (Fig. 24a) [240]. An in-situ reaction between Na metal and an *α*-Fe_2_O_3−*x*_F_*x*_ coating layer on Na_3_Zr_2_Si_2_PO_12_ results in an interphase with an outer layer of Fe and NaF and an inner layer rich in Fe^2+^/Fe^3+^ (Fig. 24b) [241]. The outer layer inhibits subsequent reactions at the Na/SSE interface, while the inner layer helps Na storage, preventing further dendritic propagation. Advanced atomic-scale methods, including high-resolution transmission electron microscopy (HRTEM), have provided compelling evidence for structural evolution of electrode/SSE interphases. For example, in-situ HRTEM was exploited to directly observe the sodiation/desodiation process of Na_3_V_2_(PO_4_)_3_ cathode, revealing the formation of an intermediate Na_2_V_2_(PO_4_)_3_ phase from the changes of lattice spacings from 2.992 to 2.542 Å that promotes interfacial stability. The gradual volume changes and reversible lattice distortions captured at atomic resolution demonstrate a self-accommodating mechanism that maintains electrode/SSE contact under cycling [242]. Similarly, in-situ HRTEM was also used to track the diffusion of precursor NaH_2_PO_4_ into cathode Na_3_V_2_(PO_4_)_3_ (NVP) on Na_3_Zr_2_Si_2_PO_12_ ASSNIBs [243]. HRTEM and the corresponding fast Fourier transforms (FFTs) patterns demonstrated NVP epitaxially grew with the precursors NH_4_VO_3_ and NaH_2_PO_4_ at the atomic scale, demonstrating that epitaxial growth processes significantly improved interfacial cohesion and reduced stress-induced degradation. The results highlighted that controlled precursor diffusion and crystallographic alignment can tailor interfacial structures for enhanced mechanical and electrochemical stability [243]. Combining these studies, the critical role of atomic-scale characterisation was demonstrated in guiding interfacial engineering to enhance structural stability of materials and electrochemical performance of ASSNIBs. Moreover, atomic layer deposition (ALD) facilitates precise control of thin-film coatings in terms of thickness, composition and uniformity at atomic-level precision, addressing challenges related to interface stability. The deposition of TiO_2_ film on Na_3_Zr_2_Si_2_PO_12_ SSE using ALD resulted in a stable and conductive TiO_2_ interphase (Fig. 24c). XPS confirmed the formation of this beneficial layer, showing its capability to greatly improve Na^+^ mobility. Further supported by DFT calculations, it was revealed that the TiO_2_ interphase significantly strengthens the interaction between Na metal and the solid electrolyte, markedly enhancing wettability and lowering interface resistance. Additionally, the electrostatic potential formed at the interface considerably reduces electronic conductivity, thus effectively preventing the growth of Na dendrites and promoting enhanced cycling stability [244]. TiO_2_ ultrathin films were coated on Na_0.7_MnO_2_ (NMO) cathode particles using atomic layer deposition (ALD) [245]. The NMO particles with ten cycles of TiO_2_ ALD exhibited an ionic conductivity of 0.37 mS cm^−1^ and demonstrated a higher discharge capacity of 160 mAh g^−1^ compared with non-coated NMO. The performance was maintained over 100 cycles of charge and discharge at 0.2 C with suppressed undesirable side reactions and minimised Jahn–Teller distortion in the NMO structure, highlighting the potential of ALD coatings for high-performance ASSNIBs [245]. Other strategies, such as cellulose − poly(ethylene oxide) (CPEO) interlayer [246], AlF_3_ coating layer [247], Pb/C interlayer [248], and ultrasound welding of Na/*β*-Al_2_O_3_ [249], further illustrate the diversity of interfacial engineering for Na SSEs [250–252]. In contrast, modifying the cathode/SSE interface typically involves solid-electrolyte coatings on cathode materials [253]. For instance, core–shell Fe_1−*x*_S@Na_2.9_PS_3.95_Se_0.05_ nanorods coated with Na_2.9_PS_3.95_Se_0.05_ achieve a close-contact cathode/SSE interface (Fig. 24d). Similarly, Na_3_PS_4_ coating on NaCrO_2_ [85] and Mo_6_S_8_ [254] cathodes with solution-based method also reduces interfacial resistance [85]. In addition, in-situ preparing composite cathode material as a work in Na_2_S-Na_3_PS_4_-CMK-3 composite cathode is also demonstrated as an effective method for optimising cathode/SSE interface [255].

## Summary and Prospectives

In this review, we summarise the state-of-the-art Na SSEs, categorising them into oxides, sulfides, and halides, along with their crystal structures, ion conduction mechanisms, and electrochemical performance. Subsequently, we discuss key challenges in Na SSEs, such as low ionic conductivity, unstable electrode/electrolyte interfaces, and high material costs. To deepen the understanding of these issues, we emphasise advanced characterisation and modelling techniques, including those essential for probing spatially resolved Na-ion transport, real-time interfacial evolution, and localised degradation processes, and comparing with conventional electrochemical tests, structural characterisation and modelling methods. Finally, building upon the insights gained from the advanced characterisation and modelling techniques into Na-ion conduction mechanisms and interfacial dynamics, we introduce three promising strategies for material improvement: microstructural design, mixed-ion strategies, and interface engineering.

Since the 1960s, various Na SSE families have been developed, such as *β*-Al_2_O_3_-type Na_2_O·*x*Al_2_O_3_, NASICON-type Na_1+*x*_Zr_2_Si_*x*_P_3−*x*_O_12_, Na_3_PS_4_, Na_11_Sn_2_PS_12_, anti-perovskite Na_3_OCl and metal-centred Na_2_ZrCl_6_, each exhibiting distinct electrochemical behaviours. For instance, Na_3_PS_4_ and Na_11_Sn_2_PS_12_ SSEs demonstrate high ionic conductivities exceeding 1 mS cm^−1^, while halide SSEs such as Na_2_ZrCl_6_ offer superior oxidative stability with high-voltage cathodes (approaching 4 V vs. Na/Na^+^). Nonetheless, common drawbacks persist across these materials, including the limited conductivity of halides, cathode/SSE incompatibility, dendritic Na growth, and reliance on costly or rare elements. To better understand the underlying conduction and interfacial behaviours of Na SSEs, conventional characterisation techniques, such as CV, EIS, DC polarisation, XRD, solid-state NMR and XPS, alongside modelling methods including BVSE, DFT and AIMD, have been widely employed. However, these methods often provide only static or indirect insights and may fall short under realistic operating conditions. To overcome these limitations, advanced characterisation techniques have been introduced. Cryo-electron microscopy enables nanoscale imaging of sensitive interfaces; in-situ and operando methods capture dynamic structural and chemical evolution during cell operation; and machine learning-accelerated simulations offer predictive insights into ion transport and interfacial mechanisms across large chemical spaces. Based on these insights, efforts have increasingly focused on improving Na SSE performance through targeted strategies. Microstructural design and mixed-ion approaches have shown promise in mitigating dendrite formation and enhancing ionic conductivity, while interface engineering has been employed to lower interfacial resistance and stabilise the electrode/SSE interface.

Despite rapid advancements in Na SSEs and related devices, further research is needed to refine the rational design of Na SSEs. First, theoretical tools like DFT and AIMD can offer critical insights into factors such as band structures, structural stability, and Na-ion migration pathways [256]. DFT calculations can predict band structures [257] and formation energies [258] of various crystal frameworks, thus assisting the rational design of electrochemically and structurally stable Na SSEs. AIMD simulations, meanwhile, provide a dynamic view of Na-ion behaviours, facilitating the screening of potential materials and guiding experimental validation, thus accelerating the development of high-performance Na SSEs [82]. However, the high computational cost of AIMD still poses practical limitations, particularly at low temperatures, as simulations must often be confined to small supercells and elevated temperatures (> 500 K) to ensure adequate diffusion statistics. Machine learning is poised to further revolutionise accelerated electrolyte discovery, enabling high-throughput screening of mixed-anion systems, property prediction and mechanistic interpretation across complex chemical spaces to identify novel compositions beyond current trial-and-error approaches by learning from datasets generated from DFT, AIMD, or experimental measurements. Machine learning has already been used in the studies of argyrodite [177], Na_3_PS_4_ [175] and NASICON [176] SSEs. The recent studies demonstrate its promise in addressing critical bottlenecks, such as identifying stable compositions, predicting ionic conductivity and understanding Na-ion transport mechanisms at the atomic scale. In one notable approach, a comprehensive screening of 4 375 hypothetical Na-based argyrodite structures was performed using a combination of DFT, AIMD and graph-based machine learning models [177]. Key properties relevant to electrolyte performance, such as formation energy, energy above the convex hull, bandgap and electrochemical stability window, were computed and validated using high-fidelity datasets. A multi-objective Pareto sorting scheme was employed to select the most promising compositions, followed by conductivity estimation using AIMD. To ensure reliability, the competing decomposition products for stability evaluation were carefully curated by combining Materials Project, Inorganic Crystal Structure Database (ICSD), Google DeepMind databases and recalculated under a unified DFT protocol, achieving a single-fidelity dataset for energy above hull and electrochemical window predictions. To further enhance computational efficiency and reliability, two advanced graph neural networks, Connectivity Optimized Graph Network (coGN) and its nested variant coNGN, were used to predict material properties. The coGN architecture efficiently exploited symmetry operations through asymmetric unit cells, minimising redundant calculations, while coNGN incorporated nested line graphs to capture bond-angle-dependent features, offering better performance for complex crystal systems despite increased computational demands. This innovation allowed regression tasks for thermodynamic and electrochemical properties to reach state-of-the-art benchmark accuracies. In addition, these models represent crystal structures as graphs and learn structural-property relationships from training data without requiring handcrafted features. Their predictive accuracy rivalled DFT for multiple target properties while dramatically reducing computational cost, making them ideal for high-throughput electrolyte screening. Looking ahead, coupling these predictions with experimental and theoretical feedback loops offers a promising route to expand the design space and expedite the discovery of next-generation Na SSEs.

To advance the field, real-time in-situ and operando advanced characterisation techniques of Na SSE and ASSNIBs are essential, as they enable continuous monitoring of dendrite growth and interfacial changes under practical cycling conditions, unlike conventional ex-situ methods that capture only static snapshots [164]. These dynamic insights are crucial for identifying early-stage failure processes and building a more complete mechanistic understanding for the rational design of stable Na SSEs and interfaces. Uncovering the mechanisms governing Na dendrite formation and interfacial degradation at the cathode/SSE boundary is vital for the development of stable ASSNIBs. Observations from Li-based systems indicate that dendrite growth involves distinct initiation and propagation phases [150], which can also be used to guide the construction of ASSNIBs. Recent multiscale studies on *β*″-Al_2_O_3_ electrolytes reveal that Na dendrite advancement is driven by repeated cycles of metal deposition and fracture. Cracking occurs along Na^+^ transport planes, creating pathways that facilitate further metal growth, with SEM and T_2_ weighted contrast maps confirming the presence of stress-induced delamination and conduction channel closure during cycling [155]. Such findings suggest that electrochemical and mechanical effects are coupled, and that relieving or preventing this coupling could limit dendrite progression. One strategy under investigation involves electrolyte modification, for instance, incorporating LaCl_3_ into Li_6_PS_5_Cl to passivate the interface and reduce crack propagation risks [259]. Likewise, controlling mechanical parameters, such as applying lower stack pressures, may hinder the propagation stage of dendrites, as recently demonstrated in operando studies of Li systems [150]. Complementing the chemically resolved analysis, operando synchrotron X-ray tomography has proven highly effective in visualising internal structural changes in SSEs during battery operation [260]. In the case of Ti-doped NASICON electrolyte, tomography was employed to monitor morphological evolution under galvanostatic cycling. The method provided 3D imaging at micrometer resolution, allowing capturing dynamic changes in the pore network of SSE. Initially, Na plating and stripping processes were found to be reversible, but prolonged cycling led to irreversible pore growth, densification, and accumulation of inactive Na within the structure. These structural changes coincided with the rising cell impedance, consistent with a degradation mode driven by pore-filling. Such detailed spatial mapping would be difficult to achieve using conventional techniques, emphasising the unique advantages of advanced characterisation such as operando synchrotron tomography for diagnosing internal failure pathways. Together, operando advanced characterisation methodologies can capture the real-time evolution of battery materials, providing a deeper understanding of failure mechanisms, interfacial dynamics and structural evolution in ASSNIBs. Looking ahead, integrating such advanced characterisation with modelling and targeted material design will be key to suppressing both dendrite initiation and propagation, and to realising long-lasting, scalable ASSNIBs.

Quantitative mechanical assessments, such as nanoindentation studies, are promising for understanding the Na dendritic growth in ASSNIBs [261]. A good example is the nanoindentation study in the NASICON-type Na_1+*x*_Mn_*x*/2_Zr_2−*x*/2_(PO_4_)_3_, which demonstrated the correlation between Na content and the hardness of SSE [178]. Given the significant influence of mechanical properties on resistance to dendrite initiation and propagation, further investigations employing nanoindentation techniques will provide deep insights into the correlation between SSE structure, interfacial properties and mechanical resilience. Such studies will be valuable in developing a more comprehensive understanding of how microstructural optimisation and interfacial engineering contribute to dendrite resistance mechanisms and stability of ASSNIBs.

It is important to recognise that comprehensive quantitative analysis, such as lifecycle assessments (LCAs) examining Na SSEs and cost analysis of different synthesis routes, remain scarce in current studies of Na SSEs [262]. Future research should aim to address this gap by developing quantitative frameworks engaging in comparing the lifecycle impact of materials, synthesis energy and time requirements, along with recyclability across different Na SSEs. Such quantitative assessments would greatly enhance the understanding of sustainability, economic feasibility and practical scalability of Na SSEs.

To enhance comparability and reproducibility across studies on Na SSEs and corresponding ASSNIBs, it is essential to standardise electrochemical testing protocols. Differences in testing conditions, such as pellet densities, applied stack pressure or measurement protocols, can influence reported ionic conductivities. Recently, a benchmarking study demonstrated significant variability in battery performance arising from inconsistent cell assembly conditions, highlighting the critical need for establishing a uniform standard in SSB testing protocols (e.g., stack pressures, pressing durations and impedance measurement conditions) [263]. Moving forward, implementing standardised protocols would substantially advance the consistency and reliability of SSB testing results, facilitating the rapid development of ASSNIB technologies.

Lastly, advanced fabrication techniques such as ALD and 3D printing present exciting opportunities for constructing Na SSEs directly on electrode surfaces [264], allowing precise control over SSE thickness and enhancing interfacial contact. By reducing interfacial resistance and tailoring electrolyte structures to match electrodes, these techniques can significantly boost ionic transport and wettability at the electrode/SSE interfaces, paving the way for scalable, high-efficiency Na SSEs in next-generation energy storage applications.
